# Multi-Tasking Role of the Mechanosensing Protein Ankrd2 in the Signaling Network of Striated Muscle

**DOI:** 10.1371/journal.pone.0025519

**Published:** 2011-10-10

**Authors:** Anna Belgrano, Ljiljana Rakicevic, Lorenza Mittempergher, Stefano Campanaro, Valentina C. Martinelli, Vincent Mouly, Giorgio Valle, Snezana Kojic, Georgine Faulkner

**Affiliations:** 1 Muscle Molecular Biology Group, International Centre for Genetic Engineering and Biotechnology, Trieste, Italy; 2 Institute of Molecular Genetics and Genetic Engineering, University of Belgrade, Belgrade, Serbia; 3 Centro Ricerche Interdipartimentale Biotecnologie Innovative, University of Padova, Padova, Italy; 4 Institut de Myologie, UM76, University Pierre et Marie Curie, Paris, France; Inserm U869, France

## Abstract

**Background:**

Ankrd2 (also known as Arpp) together with Ankrd1/CARP and DARP are members of the MARP mechanosensing proteins that form a complex with titin (N2A)/calpain 3 protease/myopalladin. In muscle, Ankrd2 is located in the I-band of the sarcomere and moves to the nucleus of adjacent myofibers on muscle injury. In myoblasts it is predominantly in the nucleus and on differentiation shifts from the nucleus to the cytoplasm. In agreement with its role as a sensor it interacts both with sarcomeric proteins and transcription factors.

**Methodology/Principal Findings:**

Expression profiling of endogenous Ankrd2 silenced in human myotubes was undertaken to elucidate its role as an intermediary in cell signaling pathways. Silencing Ankrd2 expression altered the expression of genes involved in both intercellular communication (cytokine-cytokine receptor interaction, endocytosis, focal adhesion, tight junction, gap junction and regulation of the actin cytoskeleton) and intracellular communication (calcium, insulin, MAPK, p53, TGF-β and Wnt signaling). The significance of Ankrd2 in cell signaling was strengthened by the fact that we were able to show for the first time that Nkx2.5 and p53 are upstream effectors of the *Ankrd2* gene and that Ankrd1/CARP, another MARP member, can modulate the transcriptional ability of MyoD on the *Ankrd2* promoter. Another novel finding was the interaction between Ankrd2 and proteins with PDZ and SH3 domains, further supporting its role in signaling. It is noteworthy that we demonstrated that transcription factors PAX6, LHX2, NFIL3 and MECP2, were able to bind both the Ankrd2 protein and its promoter indicating the presence of a regulatory feedback loop mechanism.

**Conclusions/Significance:**

In conclusion we demonstrate that Ankrd2 is a potent regulator in muscle cells affecting a multitude of pathways and processes.

## Introduction

For any cell it is important to respond to external stimuli as quickly and efficiently as possible, this is especially true for striated muscle cells that are subjected to a variety of stress on a continuous basis. In striated muscle focal points for mechanotransduction are found at the Z-disc, the Z-disc/I-band interface and the M-band, the link between them being the giant protein titin that spans the sarcomere from the Z-disc to the M-band [1]. A signal complex sensitive to mechanical stress (such as stretch and muscle injury) is located at the I-band of the sarcomere and assembled on the N2A region of titin. Titin serves as a scaffold for the organization of the signal complex composed of myopalladin, calpain 3 and the muscle ankyrin repeat proteins (MARPs) [1], [2]. The MARP family of proteins is composed of Ankrd1/CARP [3], [4], [5], Ankrd2 [6], [7] also known as ARPP [8] and DARP [9]. These proteins are located at the Z/I band interface and are expressed both in cardiac and skeletal muscle, however Ankrd1/CARP is expressed primarily in cardiac muscle [3], [4], [5] and Ankrd2 mainly in skeletal muscle [6], [7], [8]. The MARPs have several important functional domains: ankyrin repeats involved in protein-protein interaction, PEST motifs that are regions of protein instability and putative nuclear localization signal (NLS) for sorting proteins into the nucleus [1].

To study the role of the MARP proteins in skeletal muscle Barash and colleagues produced mice with either single, double or triple knockouts of these members [10]. However these animals showed only minor differences in fiber size and type compared to wild type mice, with a trend towards a slower fiber-type distribution. In triple knockout mice, after eccentric contractions, slight differences in mechanical behavior were observed, and both MyoD and muscle LIM protein were up-regulated [10]. Although MARP knockout mice showed a relatively mild phenotype, the MARP proteins are important for normal function of striated muscle. In fact, Ankrd1/CARP mutations have been implicated in dilated cardiomyopathy (DCM) probably due to the disruption of its binding to Talin-1 and FHL2 (four and a half LIM domains 2) which could cause dysfunction of the cellular stretch-based signaling machinery [11]. Also Ankrd2 expression is altered in some skeletal muscle disorders: it is down-regulated in patients with muscular dystrophy, while up-regulated in atrophic or damaged myofibers in patients with congenital myopathy. In spinal muscular atrophy Ankrd2 is induced in hypertrophic myofibers and Ankrd2–positive myofibers are arranged in groups as a result of the process of denervation [12].

Ankrd2 is thought to have dual, structural and signaling roles, and could link the elastic I-band region as a stress sensor for transcriptional control in the nucleus. Its stretch sensor function has already been demonstrated [6] and notably, in skeletal muscle it is strongly up-regulated under acute stress such as muscle stretch [6], injury [13], denervation [14] and differentiation [7], [15]. After muscle injury Ankrd2 accumulated in the nuclei of myofibers adjacent to the damaged ones [13]. Ankrd2 can also be found in the nucleus of proliferating myoblasts where it may regulate the expression of specific target genes by acting as a transcriptional co-factor since it binds to and modulates the activity of transcription factors (TFs) p53, YB-1 and PML [15]. It has been suggested that the modulator protease calpain 3 regulates sarcomeric localization of MARPs and their interactions with other proteins of the signaling complex. Both Ankrd1/CARP and Ankrd2 are digested by calpain 3 [2], [16] and as demonstrated for Ankrd1/CARP calpain 3-mediated cleavage strengthens its interaction with titin N2A region [16]. Apart from titin and calpain 3, Ankrd2 also interacts with the Z-disc protein telethonin that enables precise and rigid anchoring of titin within the sarcomere [15].

Currently, little is known about muscle specific regulation of Ankrd2 expression. Bean and colleagues have shown that Ankrd2 expression is induced by MyoD, a key regulator of myogenic differentiation [17]. Ankrd2 contributes to the coordination of proliferation and apoptosis during myogenic differentiation *in vitro*, possibly via the p53 network, as p53-activated apoptosis was promoted in C2C12 myoblasts overexpressing Ankrd2. Also, both MyoD and late markers of differentiation were downregulated whereas Ankrd2 silencing resulted in proliferation of mouse myoblasts [18]. Recently, Mohamed and colleagues demonstrated that, depending on the stretch direction, Ankrd2 expression could be up-regulated either by activation of the NFkB or AP-1 signaling pathways [19]. The transcription factor nuclear factor-kappaB (NF-κB) is particularly interesting since it is activated by mechanical stretch [20] and implicated in regulation of muscle atrophy. Recently, Ankrd1/CARP was identified as indirect target gene of two transcription factors p50 and Bcl-3, shown to be required for muscle disuse atrophy [21]. The classical NF-κB pathway has a role in skeletal muscle cells differentiation and acts to prevent their premature differentiation [22].

The localization of Ankrd2 in the I-band of muscle as part of a putative mechanosensing complex [1], its accumulation in the nucleus after muscle injury [13] and in proliferating myoblasts [15], together with its interaction with transcription factors (p53, YB-1 and PML) [15] and its localization in euchromatin [13], strongly supports Ankrd2 role in the regulation of gene expression. The aim of this work was to discover pathways in which Ankrd2 has a pivotal role by identifying potential targets of Ankrd2, as well as regulators of Ankrd2 expression to bridge current gap in knowledge related to Ankrd2 biological functions and its regulatory role in muscle.

## Results

In order to discover the cellular networks and pathways in which Ankrd2 plays an active role, we employed microarray technology to look at the gene expression profile in primary human myotubes after silencing Ankrd2 using RNA interference.

### Expression profiling of endogenous Ankrd2 silenced myotubes

To determine genes and ultimately pathways affected by silencing Ankrd2 in human differentiated muscle cells we used a strategy exploiting Adeno–associated viruses as detailed in the Experimental Procedures section. This strategy was used as differentiated muscle cells are notoriously difficult to transfect. To identify the Ankrd2 related genes involved in the crucial steps of the myogenic program a series of DNA microarray experiments were performed using total RNA from silenced and non-silenced human skeletal muscle cells (CHQ5B). In cells infected with AAV-shRNAex1-2 (S) the endogenous Ankrd2 is significantly reduced both at the RNA and protein level compared to its levels in non-silenced cells infected with AAV-shLuc (N) and uninfected control cells (C) (Figure S1).

Alterations in the transcriptional profile of Ankrd2-silenced cells compared to non-silenced cells were determined using the Whole Human Genome Oligo Microarray system (Agilent Technologies). The data were analyzed using several tools for data filtering and normalization in order to select a discrete number of differentially regulated genes with a threshold level for False Discovery Rate (FDR) 0%. Normalized expression values were used as input for the Significance Analysis of Microarray (SAM) software. Setting the delta value at 1.212, the FDR of the selected genes was equal to zero and after removing genes represented in the array by more than one spot and false genes SAM extracted 1,891 significantly differentially expressed genes. Expression value ratios (S/N) between the two channels are given as a logarithmic scale base 2 (log_2_). A threshold for differential expression of log_2_ ratio >0.8 or <−0.8 was used giving 732 single genes selected by SAM in silenced cells. However after removing genes not noted in the human gene database GeneCards v3 (http://www.genecards.org/),599 single genes were selected of which 281 were under-expressed (Table S1) and 318 were over-expressed (Table S2). As expected the Ankrd2 gene was one of the most significantly down-regulated genes with a log_2_ ratio equal to -2.02 (Table S1).

How much is gene expression altered by infection per se? In order to evaluate the impact of infection on the behaviour of skeletal muscle cells, hybridization experiments between non-silenced cells infected with AAV-shLuc and uninfected cells were performed under the same conditions as previously used for silenced versus non-silenced cells. From SAM analysis there was no marked difference between these conditions, in fact with a FDR 0% and after the elimination of repeated genes there were only 13 genes with a significant change in expression (log_2_ ratio values <−2.5). The complete list of significantly changed genes, including those identified with a higher FDR value of 5% is reported in Table S3. The vast majority of these genes are related to cell cycle or mechanisms for DNA repair and replication.

To obtain an overall view of the effect of silencing Ankrd2 in human myotubes on cellular pathways the differentially expressed genes (Tables S1 and S2) were checked by the KEGG pathway database (http://www.genome.jp/kegg/pathway.html) using the Homo sapiens reference pathway. Only pathways with more than 7 differentially expressed genes have been listed in Tables 1 and S4 but in fact many more pathways were detected by KEGG. Table S4 lists not only the genes differentially expressed in the various pathways but also contains extra information including description and log_2_ ratio, whereas Table 1 is a reduced form of this information only listing gene symbols and if up- and down- regulated. The 18 pathways with at least 7 differentially expressed genes are listed in Tables 1 and S4, in brackets are the number of genes whose expression has changed in each pathway: Metabolic (34); Cancer (17); Focal adhesion (14); MAPK signaling (13); Cytokine-cytokine receptor interaction (12); Regulation of the actin cytoskeleton (12); Insulin signaling (12); Wnt signaling (10); Calcium signaling (9); Gap junction (9); Hypertrophic cardiomyopathy, HCM (8); Dilated cardiomyopathy, DCM (7); Chronic myeloid leukemia (7); Endocytosis (7); Huntington's disease (7); p53 signaling (7); TGF-β signaling(7) and Tight junction (7).

**Table 1:** KEGG pathways differentially expressed in Ankrd2 silenced myotubes.

KEGG pathways	Upregulated genes	Downregulated genes
**hsa01100** **Metabolic pathways**	B3GALT4, CBS, DHRS3, GBE1, GCS1, KHK, NDST1, NDUFC2, PCK2, PLCB4, PNPLA3, POLR2K, SCA4MOL, SQLE, UAP1	ADSSL1, AKR1B10, AMPD1, AMY1C, ATP6V1E2, BCAT1, CKM, CYP27A1, GCNT3, GLUL, HADH, MLYCD, PFKM, PIK3C2B, PLCD4, PPT1, SPTLC3, ST8SIA5, TRIT1
**hsa05200** **Pathways in cancer**	ITGA6BCR, CCND1, CDK6, FGF2, JUP, MAP2K1, PDGFB, PDGFRB, RUNX1, TFG, TGFB2	ARNT, CYCS, EGLN3, FZD4, LAMA4
**hsa04510** **Focal adhesion**	ACTB, ACTN4, CAV1, CCND1, CCND2, FLNB, ITGA6, MAP2K1, PDGFB, PDGFRB, SHC2	ITGB8, LAMA4, MYL5
**hsa04010** **MAPK signaling**	BDNF, FGF2, FLNB, GADD45B, MAP2K1, MAP3K7, MKNK2, NTF3, PDGFB, PDGFRB, TGFB2	MEF2C, RPS6KA5
**hsa04060** **Cytokine-cytokine receptor interaction**	CCL2, EPOR, NGFR, PDGFB, PDGFRB, TGFB2, TNFRSF11B, TNFRSF12A, TNFRSF25	ACVR1, IL17B, IL6R
**hsa04810** **Regulation of actin cytoskeleton**	ACTB, ACTN4, ARHGEF4, ARPC1B, BAIAP2, FGF2, ITGA6, MAP2K1, PDGFB, PDGFRB	ITGB8, MYL5
**hsa04910** **Insulin signaling**	MAP2K1,MKNK2, PCK2, PRKAA2, PRKAG2, PTPRF, PYGB, SHC2	PHKG1, PPARGC1A, PYGM, RPS6KB1
**hsa04310** **Wnt signaling**	CCND1, CCND2, MAP3K7, NFAT5, PLCB4, SFRP4	DAAM1, FRAT2, FZD4, MMP7
**hsa04020** **Calcium signaling**	ADRA1B, ADRB2, PDGFRB	ADCY3, ATP2A1, PHKG1
**hsa04540** **Gap junction**	MAP2K1, PDGFB, PDGFRB, PLCB4, TUBB, TUBB3, TUBB8	ADCY3, TUBA8
**hsa05410** **HCM**	ACTB, EMD, ITGA6, PRKAA2, PRKAG2, TGFB2, TPM1	TTN
**hsa05414** **DCM**	ACTB, EMD, ITGA6, TGFB2, TPM1	ADCY3, TTN
**hsa05220** **Chronic myeloid** **leukemia**	BCR, CCND1, CDK6, MAP2K1, RUNX1, SHC2, TGFB2	
**hsa05016** **Huntington's disease**	BDNF, NDUFC2, PLCB4, POLR2K	CYCS, Dynein, PPARGC1A
**hsa04115** **p53 signaling**	CCND1, CCND2, CDK6, GADD45B, IGFBP3, SERPINE1	CYCS
**hsa04350** **TGF-beta signaling**	CHRD, GDF6, SMURF2, TGFB2	ACVR1, PITX2, RPS6KB1
**hsa04530** **Tight junction**	ACTB, ACTN4, RAB3B, YES1	MYH8, MYL5, TJP2

As seen in Tables 1 and S4 several signaling pathways were affected by silencing Ankrd2 in myotubes therefore to further investigate the regulatory role of Ankrd2 in skeletal muscle cells, we screened for proteins that interact with Ankrd2 and that could participate in signaling pathways.

### Ankrd2 can interact with PDZ-motif and SH3 domain proteins involved in signaling pathways

The results obtained from Ankrd2 silencing in human myotubes suggesting its role in intracellular and intercellular communication strongly corroborate a regulatory role for Ankrd2 as participant in signaling pathways. Therefore we choose to screen for PDZ and SH3 proteins as they are known to be involved in signaling pathways [23] and recently the PDZ-Lim protein family has been reported to mediate signals from the nucleus to the cytoskeleton [24]. Both PDZ and SH3 domains are conserved and act as modules for protein-protein interactions. Ankrd2 contains ankyrin repeats important for protein-protein interactions, therefore we hypothesized that Ankrd2 could interact with regulatory factors that also contain other types of modules for protein-protein interactions.

**Figure 1:**
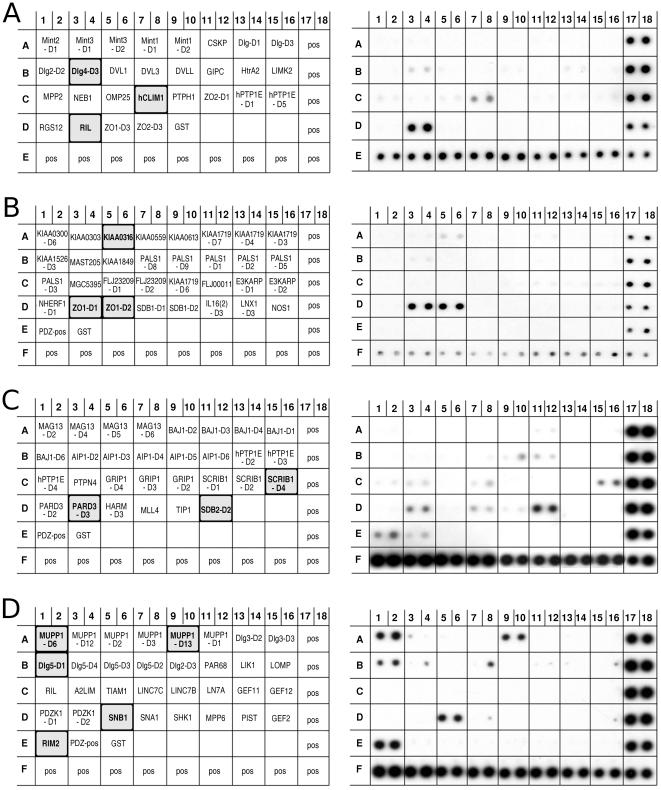
Ankrd2 can bind proteins via their PDZ domain. Panels (A), (B), (C) and (D) show respectively PDZ arrays I, II, III and IV (Panomics/Affymetrix, USA) probed with His-tagged Ankrd2 (15 µg/ml). On the left are diagrams showing of the positions of the GST-PDZ proteins on the membrane; PDZ proteins that interact with Ankrd2 are highlighted. On the right are the membranes after probing with His-Ankrd2: (A) on PDZ array I a very strong positive signal was detected for the Reversion-induced LIM protein (RIL); weak positive signals for the PDZ and LIM domain protein 1 (hCLIM1) and Discs large homolog 4 (Dlg4). (B) on PDZ array II positive signals were detected for domain 1 and 2 of Zonula occludens (ZO-1 and ZO-2). (C) on PDZ array III positive signals were detected for syntenin-2 beta, domain 2 (SDB2-D2); for partitioning-defective 3 homolog, domain 3 (PARD-3) and for Scribble domain 4 (SCRIB1-D4). (D) on PDZ array IV positive signals were detected for domains 6 and 13 of the MUPP1 protein (MPDZ, Multi-PDZ domain protein); for domain 1 of Discs large homolog 5 (DLG5-D1); for syntrophin 2 (SNTB1) and also for RIM2 (RIMS2). His-tagged ligand was spotted in duplicate along the bottom and right edge for alignment and as a positive control.

In order to identify regulatory proteins that physically interact with Ankrd2 we screened PDZ domain protein arrays (Panomics/Affymetrix, USA) with His-tagged Ankrd2 protein (Figure 1). The intensity of the spots after developing by ECL gave an indication of the binding affinity. In In Figure 1 on the left, are diagrams showing of the positions of the GST-PDZ proteins on the membrane; the PDZ proteins that interact with Ankrd2 are highlighted. On the right are the membranes after probing with His-Ankrd2. The following proteins bound strongly to Ankrd2: RIL, Reversion-induced LIM protein (Figure 1A, row D 3/4); ZO-1 D1 and ZO-1 D2, Zonula occludens (ZO) proteins (respectively, Figure 1B, row D 3/4 and row D 5/6); SDB2-D2, domain 2 of syntenin-2 beta (Figure 1C, row D 11/12); MUPP1-D6 and MUPP1-D13, domain 6 and 13 of the multiple PDZ domain protein (Figure 1D, row A 1/2 and 9/10); SNB1, Beta-1-syntrophin (Figure 1D, row D 5/6); RIM2, regulating synaptic membrane exocytosis 2 (Figure 1D, row E 1/2). Weaker positive signals could be detected for: Dlg4-D3, Discs large homolog 4, domain 3 (Figure 1A, row B 3/4); hCLIM1, PDZ and LIM domain protein 1 (Figure 1A, row C 7/8); KIAA0316, FERM and PDZ domain containing 4 (Figure 1B, row A 5/6); SCRIB1-D4, Scribble domain 4 (Figure 1C, row C 15/16); PARD-3, partitioning-defective 3 homolog, domain 3 (Figure 1C, row D 3/4); DLG5-D1, Discs large homolog 5, domain 1 (Figure 1D, row B 1/2).

**Figure 2:**
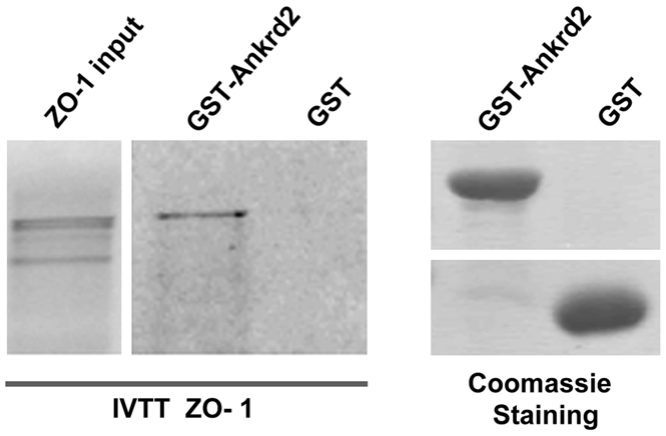
Ankrd2 interacts with tight junction protein ZO-1 (TJP1). The left panel shows GST-Ankrd2 pull down of radiolabeled ZO-1: only the GST-Ankrd2 bound to ZO-1 and not GST protein alone. GST or GST-Ankrd2 bound to glutathione-Sepharose 4B and was incubated for 3 h at RT with IVTT ^35^S ZO-1. Immobilized complexes were then washed and subjected to SDS-PAGE. The input was 10% of the total amount of IVTT ^35^S-ZO-1 was used in each binding reaction. In the right panel a SDS-PAGE gel stained with Coomassie blue shows that equal amounts of GST-Ankrd2 and GST were used in this experiment.

From the protein array data seen in Figure 1B, the Ankrd2 protein is able to bind strongly to the PDZ domains D1 and D2 of ZO-1. This interaction between Ankrd2 and ZO-1 was confirmed using an *in vitro* binding assay in which GST-Ankrd2 was used to pull-down radiolabeled ZO-1 obtained by *in vitro* transcription and translation (IVTT). In Figure 2 the right panel shows that only GST-Ankrd2 bound the IVTT ZO-1 not GST. Left panel demonstrates that equal quantities of GST-Ankrd2 and GST were used. This *in vitro* binding experiment (Figure 2) confirms the interaction detected on the PDZ membrane array (Figure 1B) between Ankrd2 and ZO-1. It is important to note that the expression of tight junction protein TJP2 (ZO-2) is down-regulated in Ankrd2 silenced cells (Table S1). Zonula occludens (ZO) proteins, ZO-1 and ZO-2 also known as Tight Junction proteins (TJP), are involved in the organization of epithelial and endothelial intercellular junctions and form a link between the junction site and the cytoskeleton by interacting directly with actin filaments [25], [26], [27].

A large number of PDZ-containing proteins have been grouped into families according to their different modular organization [28]. It is very interesting that Ankrd2 can interact with representatives from several of the PDZ-protein groups. RIL and hCLIM are members of the Enigma/PDZ-LIM family containing a N-terminal PDZ domain and one or three LIM domains. Dlg and ZO-1 are members of the MAGUK family that contain one or three PDZ domains, a SH3 domain and GUK (guanylate kinase) domain. Multi-PDZ-domain proteins, as the name suggests, contain only multiple PDZ domains. Ankrd2 can bind to MUPP1, a multiple PDZ domain protein with 13 PDZ domains, which is located at tight junction and binds to the tight junction claudins [29].

**Figure 3:**
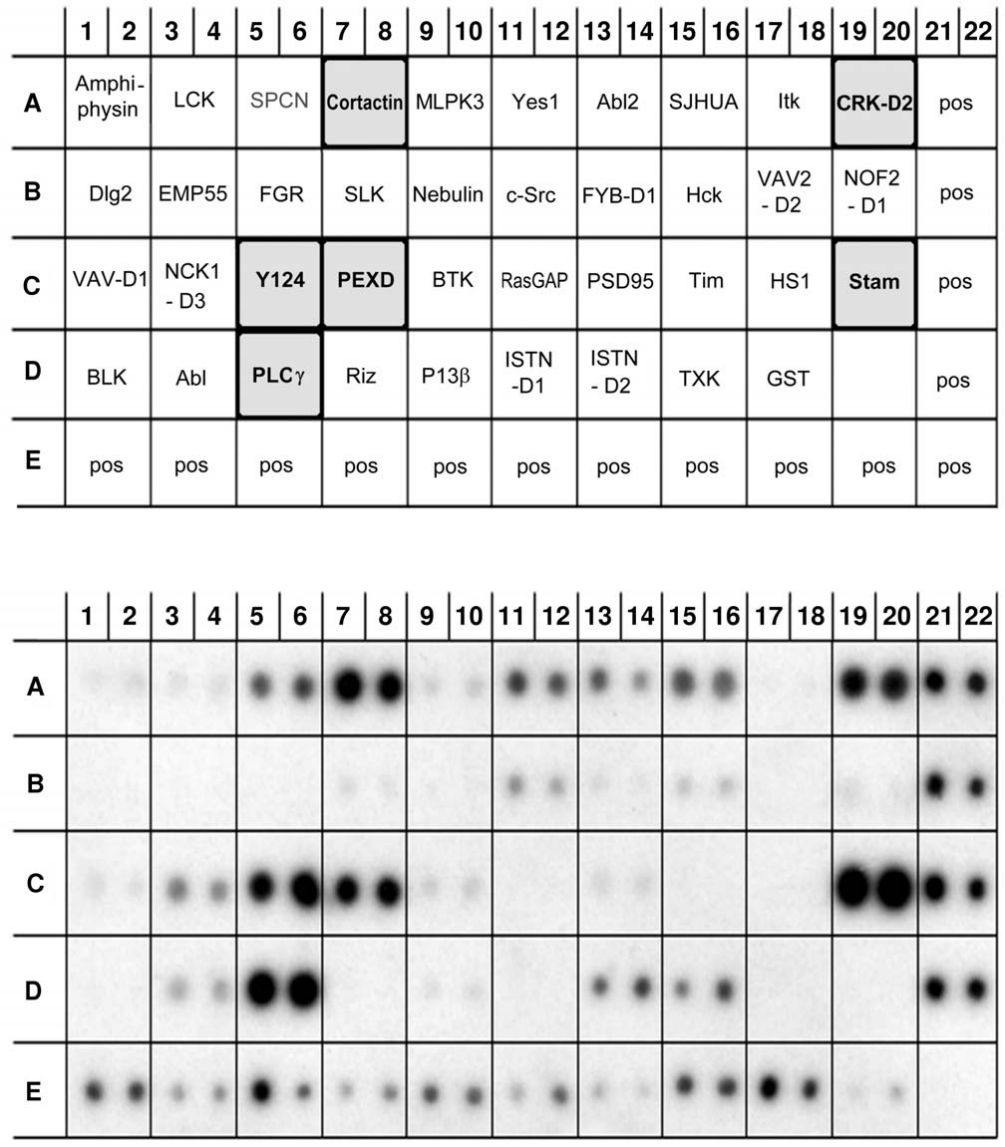
Ankrd2 can interact with proteins containing SH3 domains. A SH3 protein array (Panomics/Affymetrix, USA) was probed with His-tagged Ankrd2 protein (15 µg/ml). The upper panel is a schematic diagram of the array showing the positions of the spotted GST-proteins; proteins positive for interaction with Ankrd2 are highlighted. The lower panel shows the membrane after hybridization with His-Ankrd2 protein. Ankrd2 bound strongly to the following SH3 proteins: Cortactin, CRK-D2, Y124, PEXD, Stam, and PLCγ. The positive controls (in duplicate) intended for alignment are seen at the bottom and the right edge of the blot.

Src homology 3 (SH3) domain is a 60 amino acid protein domain that mediates protein-protein interactions by binding to proline-rich peptide sequences [30]. It is found in a large number of proteins including cytoskeletal and many intracellular signaling protein families such as the P13 kinase, Ras GTPase, CDC24 and CDC25. Computer analysis (SH3-Hunter, http://cbm.bio.uniroma2.it/SH3-Hunter/) predicted two overlapping regions (aa 107–113 and aa 110–115) of the Ankrd2 protein able to interact with SH3 domains. In order to confirm this finding a SH3 Domain Array (Panomics/Affymetrix, USA) spotted in duplicate with 38 different SH3 domain proteins was probed with His-tagged Ankrd2 protein (Figure 3). Strong positive signals were detected for the following proteins: Cortactin (row A 7/8); CRK-D2, sarcoma virus CT10 oncogene homolog, domain 2 (row A 19/20); Y124, PAK-interacting exchange factor beta (row C 5/6); PEXD, Peroxisomal membrane protein PEX13 (row C 7/8); Stam, Signal transducing adaptor molecule (row C 19/20); PLC γ, Phospholipase C gamma-1 (row D 5/6). Weaker interactions with Ankrd2 and SH3 proteins were also detected, however in order to avoid false positives only the strong signals were considered significant. Ankrd2 interacting partners containing PDZ and SH3 domains are listed in the Table 2.

**Table 2:** Ankrd2 protein interactions detected by protein-arrays.

Symbol	Protein Description	Pathway/Process
**Transcription factors binding to Ankrd2**		
**HAND2**	heart/neural crest derivatives 2	NFAT/Cardiac hypertrophy
HDC1	histone deacetylase 1	Notch; Cell cycle; TGFβ
**HOXA5**	homeobox A5	Skeletal development
HEY	Hey1, hairy/enhancer-of-split	Notch effector
HNF4G	hepatocyte nuclear factor 4γ	Maturity onset diabetes
JUN	transcription factor AP-1	MAPK
JUNB	transcription factor AP-1	MAPK
KLF12	Kruppel-like factor 12	Vertebrate development
LDB1	LIM domain binding 1	Transcription reg. by Pitx2
**LHX2**	LIM homeobox 2	Nervous system develop.
MADH3	SMAD family member 3	Wnt signaling; TGFβ
MAFK	transcription factor MafK	NRF2-med oxidative stress
MAX	MYC associated factor X	p38 MAPK
MECP2	MADS-box enhancer 2C	MAPK; Cancer
**MEF2C**	myocyte enhancer factor 2C	MAPK
**NFIL3**	IL-3 regul. nuclear factor	Immune response
NR1H2	nuclear receptor 1, H2	LXR/RXR activation
NR112	nuclear receptor subfam. 1	PXR/RXR activation
**PAX6**	paired box 6	MAPK/ERK
p53	tumor protein p53	MAPK; p53
**PDZ domain proteins binding to Ankrd2**		
hCLIM1	PDZ and LIM domain 1	Regulation of transcription
DLG4	discs, large homolog 4	Nos1/Huntington's disease
DLG5	discs, large homolog 5	Apoptosis; cell cycle
MUPP1	multiple PDZ domain	Tight junctions
RIL	PDZ and LIM domain 4	Actin stress fiber turnover
RIM2	reg. synaptic exocytosis 2	Intracell. protein transport
SDB2	syndecan binding protein 2	mTOR and NFAT pathways
SNB1	syntrophin, beta 1	nNOS signaling
ZO1	TJP1, tight junction prot.1	Tight and gap junctions
**SH3 domain proteins binding to Ankrd2**		
CTTN	Cortactin	Tight junction
CRK	proto-oncogene C-crk	MAPK; Actin cytoskeleton
PEXD	peroxisomal factor 13	Peroxisome
PLCγ	phospholipase C, gamma 1,	ErbB; Calcium
STAM	signal transducing adaptor 1	Jak-STAT; Endocytosis
Y124	ARHGEF7, Rho GEF 7	Actin cytoskeleton

### Ankrd2 is able to interact with several transcription factors

**Figure 4:**
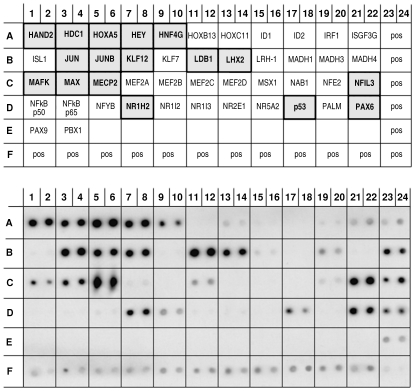
The Ankrd2 protein can interact with several transcription factors (TF). The upper panel is a schematic diagram of the Transcription Factor Array II (Panomics/Affymetrix, USA) showing the positions of the spotted His tagged TF proteins. The lower panel shows the TF protein-protein array membrane after probing with GST-Ankrd2 protein (15 µg/ml). The Ankrd2 protein bound very strongly to MeCP2 and strongly to HAND2, HDAC1, HOXA5, HEY, JUN, JUNB, KLF12, LDB1, LHX2, NFIL3 and PAX6. Weaker binding was seen with HNFG4, MAFK, MAX, NR1H2 and p53. The positive controls are at the bottom and right edge of the membranes. The TF proteins that interact with the Ankrd2 protein are highlighted.

Apart from participation in signaling pathways, Ankrd2 protein has also been suggested to regulate transcription. In fact we previously demonstrated that the Ankrd2 protein can bind three transcription factors, p53, YB-1 and PML [15]. In order to determine if other transcription factors were able to bind Ankrd2, we used a TF array (TransSignal Transcription Factor Protein Array II, Panomics/Affymetrix, USA) to screen for interaction with GST-tagged Ankrd2 protein (Figure 4). The upper panel is a schematic diagram showing the positions of 46 His-tagged transcription factors spotted in duplicate on the membrane. The lower panel shows the TF protein-protein array membrane after probing with GST-Ankrd2 protein. Ankrd2 bound strongly to several transcription factors: HAND2, heart and neural crest derivatives expressed 2 (row A 1/2); HDC1, known as HDAC1 histone deacetylase 1 (row A 3/4); HOXA5, homeobox A5 (row A 5/6); HEY, hairy/enhancer-of-split related with YRPW motif 1 (row A 7/8); Jun, v-jun sarcoma virus 17 oncogene homolog (row B 3/4); Jun B, a proto-oncogene (row B 5/6); KLF12, Kruppel-like factor 12 (row B 7/8); LDB1, LIM domain binding 1 (row B 11/12); LHX2, LIM homeobox 2 (row B 13/14); MeCP2, methyl CpG binding protein 2 (row C 5/6); NFIL3, nuclear factor, interleukin 3 regulated (row C 21/22); PAX6, paired box gene 6 (row D 21/22). Weaker binding was seen between the Ankrd2 protein and the following TFs: HNF4G, hepatocyte nuclear factor 4, gamma (row A 9/10); MAFK, v-maf musculoaponeurotic fibrosarcoma oncogene homolog K (row C 1/2); MAX, Myc associated factor X (row C 3/4); NR1H2, nuclear receptor subfamily 1, group H, member 2 (row D 7/8); p53, tumor suppressor protein (row D 17/18). The list of Ankrd2 interacting partners among transcription factors is given in the Table 2,the TF proteins that bind both the Ankrd2 protein and promoter are shown in bold. Although the Ankrd2-p53 protein interaction is weak it can be taken as positive since it had previously been confirmed by other methods [15]. However the other weak interactions need further confirmation before being taken as evidence of binding between Ankrd2 and these transcription factors.

### How does Ankrd2 interact with its binding partners?

**Figure 5:**
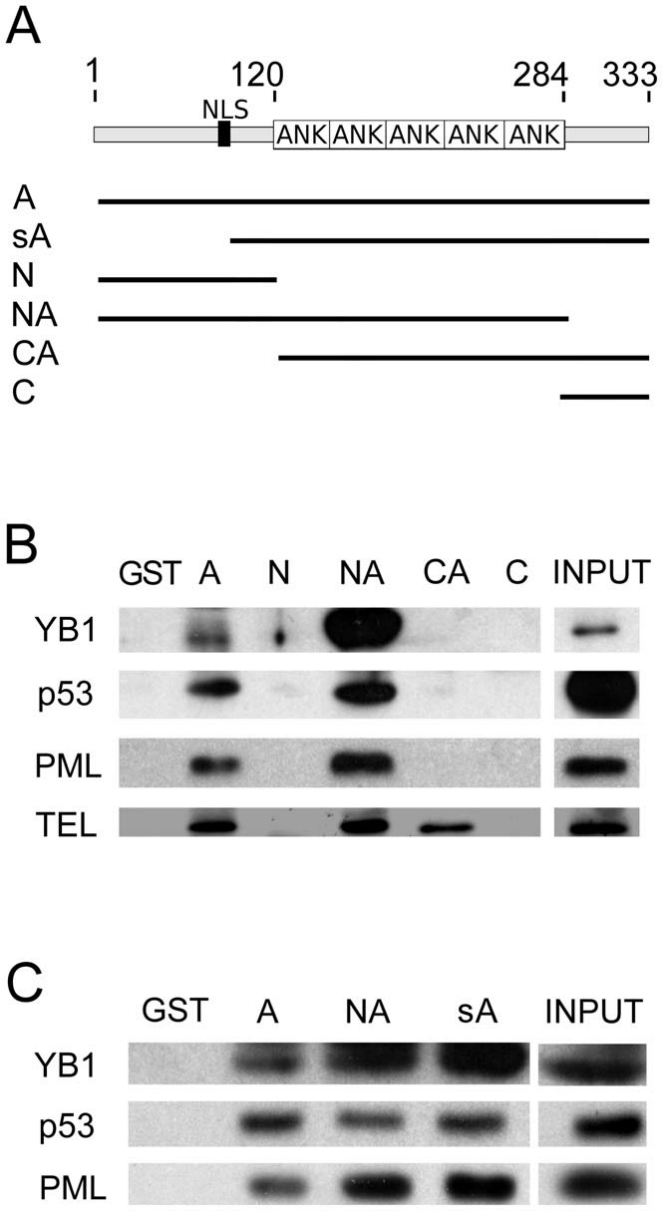
Mapping the interaction sites for YB-1, p53, PML and telethonin/Tcap on Ankrd2. (A) Diagram of Ankrd2 modular structure and deletants used in GST pull-down experiments: A, almost full length Ankrd2 protein (aa 5–333); sA, Ankrd2 protein with a 97 aa N-terminal deletion (aa 98–333); N, N-terminal (aa 5–120); NA, N-terminal plus ankyrin repeats (aa 5–284); CA, C-terminal plus ankyrin repeats (aa 121–333); C, C-terminal (aa 280–333). (B) and (C) GST pull down assays, equal amounts of GST proteins, immobilized on glutathione Sepharose (Figure S2) were mixed with cell extracts containing telethonin/Tcap, YB-1, p53 and PML. The resins were washed, subjected to SDS-PAGE and immunoblotted. Negative control is GST, positive controls (INPUT): for telethonin, 1 µg of U2OS cell lysate; for endogenous p53, 500 ng of COS7 cell lysate; for YB-1 500 ng of lysate of COS7 cell overexpressing FLAG-YB-1; and for PML, 500 ng of lysate of COS7 cells overexpressing FLAG-PML.

The fact that Ankrd2 interacts with a variety of proteins, differing both in function and cellular localization, raises the question about mechanical aspect of these interactions. Possible interaction sites are the five ankyrin repeats in its central region since these motifs are known protein interaction sites [31]. We previously demonstrated that Ankrd2 interacted with telethonin/Tcap, p53, PML and YB-1 [15]; here we mapped their binding sites on Ankrd2. GST pull down assays were performed by incubating GST-Ankrd2 and its deletants with cell lysates containing overexpressed recombinant PML, YB-1, telethonin/Tcap and endogenous p53 from COS7 cells. A schematic diagram showing the composition of the Ankrd2 protein (aa 5–333) and deletants is shown in Figure 5A: N, N-terminus (aa 5–120); NA, N-terminus and ankyrin repeats (aa 5–284); CA, C-terminus and ankyrin repeats (aa 121–333) and C, C-terminus (aa 280–333). Figure S2 shows Coomassie stained gels of these proteins demonstrating equal amounts of purified GST, GST tagged Ankrd2 and its deletants used in the GST pull down reactions in mapping experiments (Figure S2A corresponds to Figure 5B and Figure S2B to Figure 5C).

Telethonin/Tcap binds full-length Ankrd2 and also the NA and CA Ankrd2 fragments containing ankyrin repeats (Figure 5B) indicating that interaction between the Ankrd2 and telethonin/Tcap is mediated by the ankyrin repeats. Our results are in agreement with those of Hayashi and colleagues [2]; they observed a similar pattern for interaction between Ankrd2 and N2A region of titin and suggested that the second ankyrin repeat is sufficient for the Ankrd2-titin interaction. We propose that Ankrd2 is able to accomplish its interaction with sarcomeric proteins via ankyrin repeats and that these are sufficient to enable its participation in building sarcomeric multiprotein complexes. However, as can be seen in Figure 5B ankyrin repeats alone are not sufficient for interaction with the transcription factors. In order to define the specific binding sites at Ankrd2 N-terminus, a new construct sA (aa 98–333) was used. It contains ankyrin repeats and an adjacent N-terminal 22 aa region. As demonstrated in Figure 5C, the sA fragment can bind YB-1, PML and p53 suggesting that Ankrd2 N-terminal binding domain for these proteins lies in the 98–121 aa region.

### Nkx2.5 and p53 are upstream effectors of the *Ankrd2* gene but not NFkB

**Figure 6:**
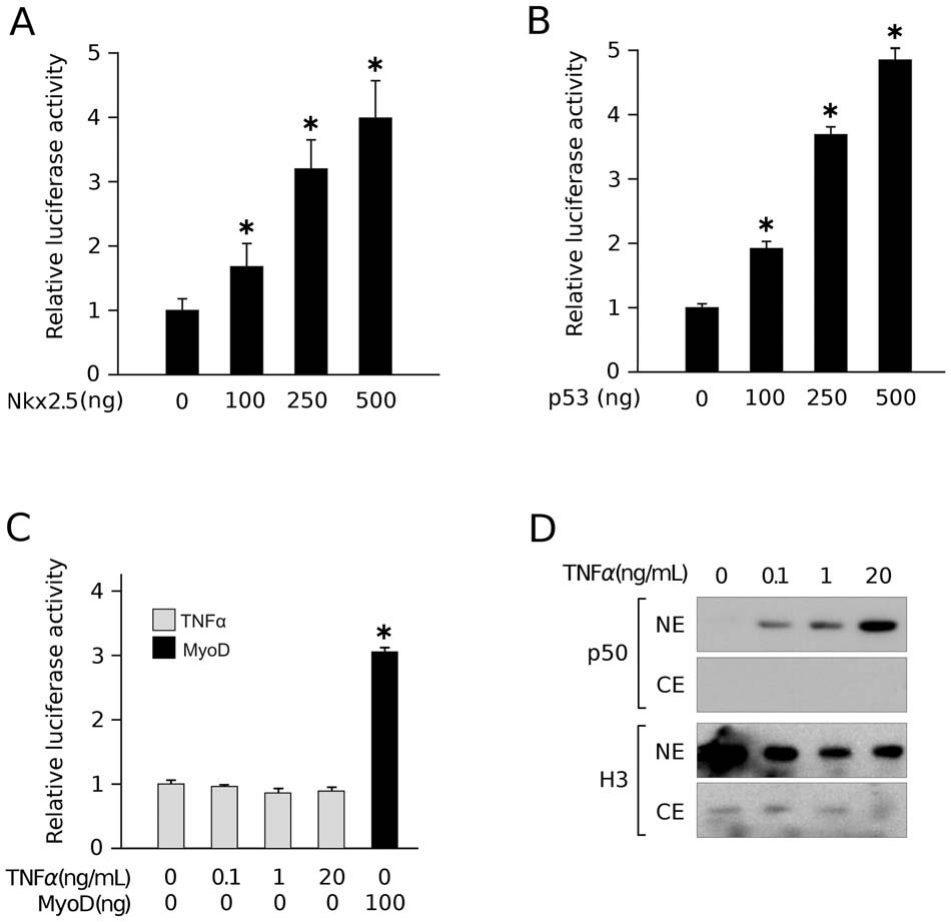
Transcriptional regulation of the *Ankrd2* promoter by Nkx2.5, p53 and NFkB. Both Nkx2.5 (A) and p53 (B) are upstream effectors of *Ankrd2* gene expression. C2C12 cells were transfected with Ankrd2 (−439/+7)-LUC and *Renilla* luciferase reporter plasmids along with increasing amounts of expression vectors for Nkx2.5 and p53 as indicated. (C) Canonical NFκB does not affect *Ankrd2* promoter activity. C2C12 were co-transfected with Ankrd2 (−439/+7)-LUC and *Renilla* luciferase plasmids and 5 hrs after transfection cells were treated with increasing amounts of TNFα in order to activate NFκB. In all of these experiments the firefly luciferase activity was normalized against the *Renilla* luciferase. The histograms show the mean of at least three independent experiments; the bars indicate the standard deviation. *p<0.05 versus control sample. (D) C2C12 cells were grown in the presence of 0.1, 1and 20 ng/ml of TNFα for 20 h and nuclear (NE) and cytoplasmic (CE) extracts prepared. Activation of NFκB by TNFα was confirmed by Western blot detection of NFκB subunit p50 in the nuclear extract (upper two panels). Efficiency of protein separation was monitored by histone H3 subcellular localization (lower two panels).

It is already known that a 280 bp of the *Ankrd2* upstream region is sufficient to confer muscle and temporal specific gene expression [7]. However computer analysis of the *Ankrd2* promoter region revealed several putative binding sites for muscle specific (MyoD and Nkx2.5) as well as ubiquitous transcription factors (p53 and NFkB). It has been demonstrated that *Ankrd2* gene expression is under the control of MyoD [17]. In order to determine if Nkx2.5 and p53 could affect the *Ankrd2* promoter, dual luciferase reporter gene assays were undertaken using an Ankrd2 (−439/+7)-LUC reporter construct. C2C12 mouse myoblasts were transiently co-transfected with Ankrd2 (−439/+7)-LUC, the *Renilla* luciferase reporter plasmid and p53-pCDNA3 or Nkx2.5-pCDNA3 expression vectors. The luciferase activity driven by the *Ankrd2* promoter increased in a dose-dependent manner, when either Nkx2.5 (Figure 6A) or p53 (Figure 6B) was expressed.

To test whether NFkB has any influence on *Ankrd2* promoter activity, C2C12 myoblasts co-transfected with Ankrd2 (−439/+7)-LUC and the *Renilla* luciferase reporter plasmids, were treated with tumor necrosis factor (TNFα) for 20 h. This cytokine activates NFkB and promotes its relocalization from the cytoplasm to the nucleus. To check if NFκB was activated under conditions used in dual luciferase assays, nuclear and cytoplasmic extracts from C2C12 myoblasts grown in the presence of different concentrations of TNFα (Figure 6D) were prepared. Subcellular localization of NFκB subunit p50 was determined by Western blot (Figure 6D). Equal amounts of nuclear and cytoplasmic extracts were subjected to SDS PAGE, immunoblotted and probed with anti-NFκB p50 and anti-histone H3 monoclonal antibodies; the latter confirmed good separation of nuclear and cytoplasmic proteins. p50 was detected exclusively in the nuclear extract; moreover a dose dependent up-regulation of p50 expression is also evident. Despite efficient activation of NFκB by TNFα, no difference in the relative luciferase activity driven from Ankrd2 promoter was detected between untreated and treated cells (Figure 6C). The discrepancy between our results and those of Mohamed and colleagues [19] could be explained by the fact that we are using different model systems. Ankrd2 is upregulated by NFkB in stretched mouse diaphragm muscle and is not a direct target of p50 suggesting that additional factors are needed in order to mediate NFkB dependent Ankrd2 expression [19]. We used unstressed mouse myoblasts and an incomplete Ankrd2 promoter therefore it is possible that additional elements are essential for NFkB dependent regulation of Ankrd2 promoter activity. In conclusion, under the conditions used only Nkx2.5 and p53 were able to modulate the *Ankrd2* promoter in myoblasts.

### The Ankrd2 promoter is able to bind several transcription factors: Hand2, HOXA5, LHX2, MECP2, NFIL3, and PAX6

Eukaryotic gene expression is regulated by transcription factors which are able to interact with specific DNA-binding elements present in gene promoters in order to modulate transcription. The activity of transcription factors is affected by a variety of factors such as cell-type, tissue specificity and the phase of the cell cycle as well as by interactions with other proteins. Knowing which transcription factors bind to the Ankrd2 promoter will allow us to understand how its expression is regulated.

**Figure 7:**
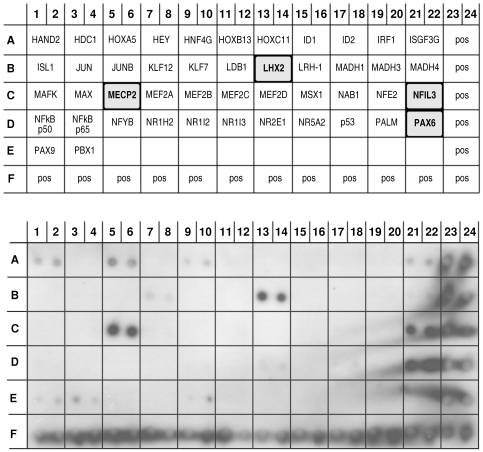
*Ankrd2* promoter DNA can interact with some transcription factors that also interact with the Ankrd2 protein. The upper panel is a schematic diagram of the Transcription Factor Array II (Panomics/Affymetrix, USA) showing the positions of the spotted His tagged TF proteins. The bottom panel shows the TF protein array membrane after hybridization with the biotinylated DNA of the *Ankrd2* promoter (−1,173 to −4 bp). The *Ankrd2* promoter DNA bound strongly to MeCP2, LHX2, NFIL3 and PAX6 and more weakly to HAND2 and HOXA5. The positive controls are at the bottom and right edge of the membranes. The TF proteins that interact with the Ankrd2 promoter are highlighted.

In order to survey multiple transcription factors a protein/DNA array was used (TransSignal Transcription Factor Protein Array II, Panomics/Affymetrix, USA) which has 46 His-tagged transcription factors spotted in duplicate on the membrane (Figure 7, upper panel). We previously used an identical membrane to screen for interactions between these transcription factors and Ankrd2 protein. To detect TF proteins that bind to theAnkrd2 promoter, the promoter DNA (from − 1,173 to −4 bp) was biotinylated, and then used to probe the array. As can be seen in Figure 7 (lower panel) transcription factors LHX2 (row B 13/14), MECP2 (row C 5/6), NFIL3 (row C 21/22) and PAX6 (row D 21/22) bound strongly to the biotinylated DNA of the *Ankrd2* promoter whereas weaker binding was observed for Hand2 and HOXA5 (row A 1/2 and 5/6, respectively). It is interesting that these six transcription factors listed in Table 3 also bound to the Ankrd2 protein (Figure 4, lower panel and Table 2, shown in bold) which would suggest that a feedback loop mechanism may be involved in controlling these interactions.

**Table 3:** Transcription factors binding to Ankrd2 promoter DNA.

Gene Symbol	Protein Description	Pathway
HAND2	Heart/neural crest derivatives 2 NFAT	Cardiac hypertrophy
HOXA5	Homeobox A5	Skeletal development
LHX2	LIM homeobox 2	Nervous system development
MECP2	MADS-box enhancer 2C	MAPK; Cancer
NFIL3	IL-3 regulatory nuclear factor	Immune response
PAX6	Paired box 6	MAPK/ERK

### Ankrd1/CARP modulates the transcriptional ability of MyoD but not of Nkx2.5 on the *Ankrd2* promoter

**Figure 8:**
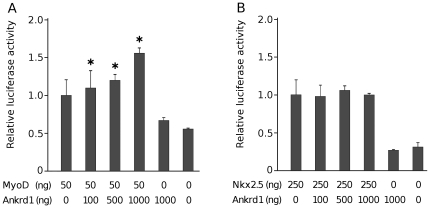
Ankrd1/CARP enhances the transcriptional ability of MyoD, but has no effect on Nkx2.5 induced up-regulation of the *Ankrd2* promoter. C2C12 were co-transfected with both Ankrd2 (−439/+7)-LUC and *Renilla* luciferase reporter plasmids as well as a constant amount of MyoD-pCDNA3 (A) or Nkx2.5-pCDNA3 (B), along with increasing amounts of an expression vector for Ankrd1/CARP, as indicated. In each assay the amount of total DNA used in transfections was kept constant by the addition of pCDNA3 vector. The firefly luciferase activity was normalized against *Renilla* luciferase. The histograms show the mean of at least three independent experiments performed in triplicate; the bars indicate the standard deviation. *p<0.05 versus control sample.

Similarly to Ankrd2 another MARP family member Ankrd1/CARP has both structural and regulatory functions in striated muscle, predominantly cardiac. Considering the regulatory role of Ankrd1/CARP as a transcriptional cofactor, its expression in skeletal muscle and the fact that recently Ankrd1/CARP was shown to enhance the transcriptional ability of p53 on the *Ankrd2* promoter [32] we examined whether it could modulate the effect of MyoD and Nkx2.5 on *Ankrd2* promoter activity. C2C12 mouse myoblasts were transiently transfected with the reporter construct Ankrd2 (−439/+7)-LUC, the *Renilla* luciferase reporter, p53-pCDNA3 or Nkx2.5-pCDNA3 as well as increasing amounts of the Ankrd1/CARP-pCDNA3 expression vectors. As can be seen in Figure 8A, Ankrd1/CARP moderately increased the transcriptional ability of MyoD emphasizing its positive effect on the *Ankrd2* promoter. However Ankrd1/CARP expression did not affect the Nkx2.*5* mediated increase of *Ankrd2* promoter activity (Figure 8B).

## Discussion

In order to study the role of Ankrd2 in cell signaling pathways we silenced endogenous Ankrd2 in human myotubes and monitored gene expression by microarray analysis. Silencing Ankrd2 expression affected genes involved in intercellular communication (cytokine-cytokine receptor interaction, endocytosis, focal adhesion, tight junction, gap junction and regulation of the actin cytoskeleton) and intracellular communication (calcium, insulin, MAPK, p53, TGF-β and Wnt signaling). Using protein arrays we identified several interacting partners of Ankrd2; PDZ- and SH3-containing proteins and transcription factors. Interestingly, the TF proteins MeCP2, Pax6, NFIL3 and LHX2 bind both to the Ankrd2 protein and Ankrd2 promoter DNA. Another novel finding was that Nkx2.5 and p53 can act as upstream effectors of the *Ankrd2* gene and that Ankrd1/CARP can affect the transcriptional ability of MyoD on the *Ankrd2* promoter. From the information obatined we can assert that Ankrd2 can act as a powerful regulator in skeletal muscle cells, affecting a multitude of pathways and processes including myogenesis, regulation of gene expression, as well as intra- and intercellular signaling. It exerts its function through interaction with transcription regulators, structural and signaling proteins. Our data are in favor of the proposed function for Ankrd2 in transmitting and transforming mechanical signals into cellular response.

From microarray profiling results, it is evident that alteration in Ankrd2 expression can cause changes in the expression of genes involved in several pathways identified using the KEGG database. Most of the affected genes belong to metabolic pathways, which is not surprising as muscle remodeling process in which Ankrd2 take a part, demands also changes in supporting energy metabolism. Apart from the collection of diverse metabolic pathways that had no particular pathway affected, there are basically three main groups of pathways with differentially expressed genes. The first group is involved in intracellular communication and affects the following signaling pathways: calcium, insulin, MAPK, p53, TGF-β and Wnt signaling. The second group is that of intercellular communication pathways affecting: cytokine-cytokine receptor interaction, endocytosis, focal adhesion (FA), tight junction (TJ), gap junction and regulation of the actin cytoskeleton. The third group is that of disease pathways including Cancer, chronic myeloid leukemia, Hungtington's disease, DCM and HCM cardiomyopathies.

In intracellular communication the majority of external signals move into the cell via ion channels, G-proteins or enyzme linked receptors. Silencing Ankrd2 affects genes in the Calcium pathway: calcium behaves as a second messenger transmitting neuromuscular activity into changes in transcription via calcineurin, calcium-dependent or calcium–calmodulin-dependent protein kinases. Interestingly, in Ankrd2 silenced myotubes FATZ-1/myozenin-1/calsarcin-2 [33], [34], [35], a calcineurin/NFAT regulator [36] is down regulated (Tables 1 and S1) whereas FATZ-2/calsarcin-1/myozenin-2 that affects fiber type composition by blocking calcineurin/NFAT activity is upregulated (Tables 1 and S2) [37].

Another important pathway affected by Ankrd2 silencing is the MAPK pathway which is activated by exercise, environmental stress as well as implicated in muscle growth and differentiation [38], [39]. The majority of the detected differentially expressed genes of the MAPK pathway are upregulated upon Ankrd2 silencing (Tables 1 and S2). Also several TF proteins that interact with the Ankrd2 protein (Table 2) are associated with the MAPK pathway: CRK, JUN, p53, MEF2C, PAX6 and MeCP2. It is noteworthy that PAX6 and MEPC2 can also bind the *Ankrd2* promoter DNA indicating the presence of control by a feedback loop mechanism.

It is interesting that one of the pathways affected by Ankrd2 silencing is the Insulin signaling pathway especially since DARP, a MARP family member, is up regulated in type 2 diabetes and thought to have a role in glucose uptake in muscle [40]. The insulin receptor substrate 1 (IRS-1) plays a key role in transmitting signals from the insulin and insulin-like growth factor-I receptor (IGF-IR) to the PI3K/Akt and Erk/MAPK pathways. Cullin7, one of the genes down regulated on silencing Ankrd2, is an E3 ubiquitin ligase that targets IRS-1 for degradation by the proteasome [41] and an increase in the IGF-IR was found to up-regulate Pax6 and glucagon which in turn activated the IRS-2/MAPK pathway that could lead to dysregulation associated with type 2 diabetes [42].

The intercellular pathways involving cell junctions are linked to the regulation of the actin cytoskeleton and cell signaling pathways. Focal adhesions act as multi-protein signaling complexes as well as having the structural role of linking membrane receptors and the actin cytoskeleton. Gap junctions are an important component of intercalated discs in cardiac muscle [43] and are necessary for skeletal muscle differentiation [44]. Tight junctions, also known as zonula occludens, are important for signaling [45]. TJ proteins ZO-1, ZO-2 and ZO-3 have PDZ and SH3 domains and link the TJ transmembrane proteins to the actin cytoskeleton [26]. Here we show that Ankrd2 can bind ZO-1 (Figure 2 and Table 2) and that ZO-2 (TJP2) is downregulated on Ankrd2 silencing (Tables 1 and S1). It is interesting that both ZO-1 [46] and Ankrd2 (Figure 3) can also bind the Src tyrosine-kinase substrate, Cortactin.

Several potential new interactions were discovered by probing arrays of PDZ, SH3 and transcription factor proteins with Ankrd2 (Figures 1, 2, 3, 4) corroborating its regulatory role. As can be seen in Table 2 some of these proteins have roles in cell junction (MUPP1, ZO1, cortactin) and signaling pathways such as TGF-β (HDC1, MADH3), Wnt (MADH3), MAPK (JUN, JUNB, MECP2, MEF2C, PAX6, p53, CRK) and NFAT (HAND2, SDB2). RIL and hCLIM are members of the Enigma family of PDZ LIM proteins that have been shown to interact with the members of both the FATZ (calsarcin/myozenin) and myotilin families of Z-disc proteins [47]. Also of note is the fact that Ankrd2 can bind to MUPP1, a multiple PDZ domain protein, known to bind the tight junction claudins [29].

Apart from the role of Ankrd2 in intracellular signaling, our results indicate a new role for Ankrd2 in intercellular signaling, in transmitting and transforming mechanical signals into cellular response. It could be hypothesized that Ankrd2 is implicated in spreading stress signals through a strictly intracellular route as well as an inside/outside path to the sarcolemma and back to the nucleus through cell-surface receptor pathways. The results obtained by DNA and protein arrays are in accordance and strongly implicate Ankrd2 role in regulatory and signaling processes.

It was demonstrated that tumor suppressor p53 has complex and multilevel interaction with MARP family members Ankrd1/CARP and Ankrd2. It behaves as an important regulator of their expression and MARPs are able to modulate the activity of p53. We have already shown their physical interaction on protein level, ability of Ankrd1/CARP to modulate p53 transcriptional activity on different promoters and potential p53 dependant regulation of Ankrd1/CARP expression through upregulation of Ankrd1 promoter activity [15], [32]. Here we show that *Ankrd2* gene expression can be regulated by p53 since it significantly increased *Ankrd2* promoter activity in luciferase assays (Figure 6B). Since in adult muscle both p53 and Ankrd2 levels increase in response to stress [6], [48] it could be suggested that p53 probably acts on *Ankrd2* gene expression in differentiated muscle cells that are exposed to stress stimuli such as stretch. Our results implicate a novel role for p53 in up-regulation of *Ankrd2* gene expression and as common regulator of MARP expression.

Alterations in interaction between Ankrd2 and p53, as well as other players in p53 pathway could be involved in pathogenesis of some tumors. In fact, the expression of Ankrd2 is elevated in a very high percentage of rhabdomyosarcomas and its use as a potential tumor marker for differential diagnosis of this soft tissue sarcoma has been suggested [49], [50]. Although there is overexpression of Wnt in embryonal rhabdomyosarcomas the canonical Wnt/B-catenin signaling pathway was down-regulated possibly due to altered AP-1 [51]. Since both Wnt [52] and Ankrd2 [13] are up regulated on skeletal muscle injury it is not surprising that several genes of the Wnt pathway are affected by Ankrd2 silencing. Apart from tumors, Ankrd2 could be linked to dystrophies and cardiac diseases since some proteins from the FATZ (myozenin/calsarcin), myotilin and Enigma families are differentially expressed in Ankrd2 silenced myotubes (Tables 1, S1 and S2).

Ankrd2 has an active role in the processes that coordinate proliferation and differentiation in muscle [18], [53]. Our results support the indispensable role of Ankrd2 in myogenesis by demonstrating that Ankrd2 silencing alters genes involved in cell to cell communication, which is very important in myogenesis. The changes in gene expression and cell morphology that occur during myogenic differentiation must be coordinated in a spatiotemporal fashion and one of the ways to achieve this is through regulation of these processes by cell-cell adhesion and resultant signaling [54]. Also, primary and secondary myoblast fusion processes require cell-cell contact [55], [56].

Ankrd2 interacts with a variety of proteins that have diverse function (structural and regulatory) and contain ankyrin repeats, modules for protein-protein interaction. Our results revealed that Ankrd2 has distinct binding patterns for its interacting partners. It uses exclusively ankyrin repeats for interaction with sarcomeric proteins (titin and telethonin), whereas N terminal domain that maps to aa 98–121 is also needed for its interaction with TFs (PML, YB-1 and p53). There are several SH3 and PDZ binding sites predicted by ELM [57] within the Ankrd2 N-terminus and although PDZ domains predominantly bind short C-terminal peptides they can also bind internal peptide sequences [58]. It is possible that binding motif(s) located in the N-terminus stabilize the interaction between Ankrd2 and regulatory proteins. On the other hand, calpain 3 could be also involved in regulation of Ankrd2 protein-protein interactions and its intracellular localization. Both Ankrd2 and Ankrd1/CARP are the substrates of this modulator protease as well as titin [2], [16]. Since the cleavage site of Ankrd2 by calpains is Arg 77 which is situated proximally from NLS, there is also a possibility that calpain 3 mediated proteolysis, apart from regulation of Ankrd2 and titin interaction, could also introduce conformational changes into Ankrd2 protein that allow differential binding of Ankrd2 to sarcomeric or regulatory proteins.

These results and observations should be analyzed in a light of the most recent result that Ankrd2 is found to be a downstream target in Akt pathway as demonstrated by Cenni and colleagues [59]. Akt-mediated signaling pathways are important in differentiation, regeneration and hypertrophy of muscle [60], [61]. It was found that Ankrd2 is a novel substrate specific for Akt2 and that oxidative stress triggers phosphorylation of Ankrd2 Ser 99 which in turn induced nuclear translocation of Ankrd2. In fact, the site of Ankrd2 phosphorylation Ser99 corresponds to Ser72 in the Ankrd2 primary sequence reported under accession number CAI14194.1 in which Arg77 is the site of calpain 3 proteolysis. This finding sheds a completely different light on these results since the sites are very close. Phosphorylation of Ankrd2 by Akt2 induces nuclear translocation of Ankrd2. The proteolysed Ankrd2 could bind more strongly to the N2A region of titin in a similar way as demonstrated for Ankrd1/CARP [16]. As phosphorylation and cleavage sites are separated by only 5 amino acids, it is possible that phosphorylation and proteolysis are competitive processes that can alter the inter-cellular distribution of Ankrd2. We hypothesize that the phosphorylated pool of Ankrd2 is predominantly located in the nuclei and that the proteolysed Ankrd2 is sequestered by the titin N2A region located at the I-band. In muscle cells that are in early phase of differentiation (binucleated cells), as well as in normal muscle tissue, both nuclear and cytoplasmic localization of Ankrd2 can be observed. Since it is known that Ankrd2 expression in the nucleus increases with stress, a possible mechanism could be that calpain 3 is not able to proteolyse Ankrd2 when Ser72 is phosphorylated, therefore Ankrd2 is not sequestered by the titin N2A region but is free to move to the nucleus. Rationalization of these separate observations on Ankrd2 selective interactions, calpain proteolysis and phosphorylation by Akt 2 kinase has yet to occur, but an association with coordination of stress response could be a possible link. The interrelation and interdependence between these three phenomena is another open question.

Molecular mechanisms that regulate *Ankrd2* gene expression and its role in the heart are completely unknown. Here we demonstrate that the cardiac specific transcription factor Nkx2.5 up-regulates the activity of *Ankrd2* promoter and that Ankrd1/CARP, a cardiac specific MARP family member, could regulate Ankrd2 expression through activation of MyoD. Apart from the well established critical role of the transcriptional activator Nkx2.5 in cardiac morphogenesis [62], it also has a role in the regulation of cardiac-specific gene expression in the adult heart. Its expression is upregulated in response to hypertrophic stimulation which may have implications in the transcriptional regulation of the cardiac gene program in hypertrophied hearts [63]. In the adult heart, Nkx2.5 also plays an important role in protecting the myocardium against cytotoxic damage [64]. Nkx2.5 mediated regulation of Ankrd2 expression in the heart could be the mechanism underlying its role in cardiac signaling pathways activated upon stress.

Although Ankrd1/CARP acts as negative co-factor in the regulation of cardiac specific gene expression [4], [65], we recently showed that Ankrd1/CARP could behave as a positive regulator of gene expression and modulate p53 activity on the *p21*, *Mdm2* and *Ankrd2* promoters [32]. Here we demonstrate that Ankrd1/CARP also acts as positive regulator of MyoD activity on the *Ankrd2* promoter (Figure 8). Therefore, apart from p53 [32], we have identified MyoD as another transcription factor whose activity can be modulated by Ankrd1/CARP. Although MyoD is known as a key regulator of skeletal muscle differentiation it was only recently detected in cardiac muscle, in periarterial Purkinje fibers [66]. Purkinje fibers are conduction cells located in the inner ventricular walls and since Ankrd2 is expressed in the ventricles [8] it is possible that the expression of Ankrd2 in cardiac muscle cells is under the control of MyoD and that Ankrd1/CARP could up-regulate MyoD dependant Ankrd2 expression in the heart. The emerging role of Ankrd2 in cardiac muscle is further supported by our finding that the HCM and DCM pathways are both affected when Ankrd2 is silenced in myotubes. One of the promising lines of future studies on Ankrd2 could be to identify mutations in Ankrd2 gene that are linked to these cardiomyopathies as has been done for Ankrd1/CARP [11], [67], [68].

It is interesting that both the *Ankrd2* promoter DNA and the Ankrd2 protein can bind transcription factors MECP2, LHX2, NFIL3 and PAX6 indicating the existence of a regulatory feedback loop mechanism (Figures 4 and 7). Transcriptional regulators HOXA5, KLF12 and LHX2 participate in developmental processes and their interaction with Ankrd2 could be important for its function in myogenesis. MECP2 is particularly interesting as a nuclear protein with a role in gene regulation. Recently it has been proposed to act not only as a transcriptional repressor but also as an activator; in fact most genes appear to be activated rather than repressed by MECP2 [69]. It should be noted that the DNA of the *Ankrd2* promoter that bound MECP2 was not methylated, however MECP2 is also capable of binding non-methylated DNA [70], [71]. MECP2 is upregulated in differentiated cardiomyocytes with a concomitant increase in global methylation and condensed chromatin [72]. The finding that Ankrd2 binds MECP2 suggests that Ankrd2 could affect not only transcription but also chromatin remodeling. Therefore, the final target of signaling cascades involving Ankrd2 could be the structural modification of chromatin.

### Conclusions

Our data support a multi-tasking role of Ankrd2 in many cellular processes regulating skeletal muscle differentiation, growth and remodeling. The results obtained from both the DNA- and protein arrays give a strong indication that Ankrd2 represents a central node within regulatory networks involved in the determination of muscle cells (MRF4), the regulation of trunk (SIX4, MEF2C) and head (PITX2, LBD1) skeletal muscle formation, control of muscle phenotype (MEF2, NFAT, JUNB, HDACs), regulation of calcineurin activity (FATZs) as well as control of muscle protein turnover (FOXO3A, PIK3C2B, NBR1, AKT signaling, FATZs). As mechano-transcriptional links in the myoblasts are found at distinct sarcomeric regions and activate different transcriptional programmes it raises the question of whether crosstalk between these pathways exists. Our data suggest that the Ankrd2 protein, itself, represents a possible link between distinct mechano-transcriptional connections. In fact, previous and current results demonstrate its functional interaction with proteins localized in the Z-disc (FATZ-1/myozenin-1/calsarcin-2, FATZ-2/myozenin-2/calsacin-1, telethonin) and M-band (NBR1 and MURFs) mechanosensing complexes. The functional significance of crosstalk between different mechanosensors and synergistic or antagonistic activation of transcriptional programmes that regulate muscle remodeling remain to be elucidated.

## Materials and Methods

### Plasmid constructs

To express Ankrd2 and its deletants, the corresponding cDNAs were inserted into the GST vector; pGEX-6P-3 (GE Healthcare). These cDNAs coded for: the full-length Ankrd2 protein (A, aa 5–333), the N-terminal (N, aa 5–120), the N-terminal with ankyrin repeats (NA, aa 5–284), the C-terminal (C, aa 280–333), the C-terminal with ankyrin repeats (CA, aa 121–333) and Ankrd2 lacking the first 97 amino acids (sA, aa 98–333). cDNAs for p53 and telethonin/Tcap were cloned into pCDNA3 (Invitrogen). PML and YB-1 were cloned into a FLAG tag vector; pCMVTag2B (Stratagene). The cDNAs for full-length Ankrd1/CARP, Nkx2.5 and MyoD were amplified by RT-PCR from human mRNA (Ambion), then cloned into pCDNA3. The proximal promoter region of the *Ankrd* gene (−439/+7) was amplified from human genomic DNA with primers, GCGACTCGAGGTACAGAACTGTCCTG and ATATAAGCTTCGCCTCTGCAGGCC, and cloned into the promoterless luciferase reporter gene vector pGL4.1 (Promega).

### Cell culture, transfections and preparation of protein extracts

COS-7 (CRL-1651), U2OS (HTB96), SaOs2 (HTB-85) and C2C12 (CRL1772) were obtained from the ATCC (Manassas, VA, USA). COS-7 cells and C2C12 mouse myoblasts were maintained in DMEM containing 10% (v/v) fetal calf serum (FCS) and gentamycin (50 µg/mL) whereas U2OS and SaOs2 cells were grown in the same medium but with 20% FCS. Primary human myoblasts CHQ5B cells were obtained and grown as described previously [33]. Differentiation medium was DMEM supplemented with 0.4% Ultroser G (BioSepra Spa, France). Cells were transfected using PolyFect (Qiagen), SuperFect (Qiagen) or *Trans*IT-LT1 (Mirus) according to the manufacturer's protocols. U2OS cells transfected with telethonin/Tcap were treated with the proteosomal inhibitor MG132 (Sigma) two hours before harvesting. Cells were harvested 24 hours after transfection, washed and then lysed in buffer containing 50 mM Hepes (pH 7.0), 250 mM NaCl, 0,1% (v/v) NP-40 and protease inhibitors (Roche). In order to activate transcription factor NFκB, C2C12 myoblasts were grown in the presence of 0.1, 1 and 20 ng/ml of TNFα (Promega) for 20 h. Nuclear and cytoplasmic extracts were prepared using ProteoJET™ Cytoplasmic and Nuclear Protein Extraction Kit (Fermentas) according to the manufacturer's instruction.

### Silencing of endogenous Ankrd2 in human myotubes

AdenoAssociated Virus (AAV) was used to deliver shRNA into primary muscle cells during differentiation. The sequence of the siRNA from *Ankrd2* exon 1–2 that reduced the expression of Ankrd2 protein in transfected COS-7 cells was used as a template to design both sense and antisense oligonucleotides (21 nucleotides). These were annealed and cloned as a ds oligo into the pZAC−U6−CMV−ZsGreen plasmid (a gift from Dr. Julie Johnston, University of Pennsylvania, USA). The pZAC−U6−CMV−ZsGreen plasmid contains a U6 promoter for RNA polymerase III transcription of shRNA and a CMV promoter for expression of the fluorescent protein ZsGreen as a control of transfection or infection. The vector used for silencing Ankrd2, AAV-shRNAex1-2, was prepared by the ICGEB Telethon Core Facility, Trieste. Since Ankrd2 is upregulated on differentiation it was necessary to infect already differentiating CHQ5B cells (after 5 days differentiation) in order to have a good level of endogenous Ankrd2 expression for silencing. Cells were harvested 4 days after infection (total of 9 days differentiation). Total RNA was extracted and analyzed by RT-PCR for Ankrd2 and GAPDH expression (Figure S1, two upper panels). The cell lysates were analyzed by Western blot for expression of Ankrd2, ZsGreen, Myosin Heavy Chain (MHC) and GAPDH (Figure S1, lower four panels). GAPDH was used as a loading control, MHC as an indicator of differentiation and ZsGreen as a control of expression of AAV-shRNAex1-2. In cells infected with AAV-shRNAex1-2 (S) the endogenous Ankrd2 is significantly reduced both at the RNA and protein level compared to its levels in non-silenced cells infected with AAV-shLuc (N) and uninfected control cells (C).

### Microarray experiments

For microarray experiments the conditions described above were used; CHQ5B cells were differentiated in low serum for 5 days and then not infected or infected with AAV-shRNAex1-2 or the control AAV-shLuc and harvested after 4 days of infection (Figure S1). Total RNA samples were subjected to retro-transcription with poly dT primers; cDNA was synthesized incorporating Cy3- or Cy5-labeled CTP. The samples were mixed (silenced with non-silenced cells and uninfected with non-silenced cells) and hybridized to the oligos of the Whole Human Genome Oligo Microarray (Agilent Technologies). After hybridization the microarray slides were scanned for acquisition of fluorescence intensity values. Total RNA from CHQ5B cells was used in seven distinct hybridizations therefore for each spot on the array there are 10 expression values (5 for silenced cells and 5 for non-silenced cells) and the differential expression was obtained from the relative abundance of hybridized mRNA. Raw expression data were normalized with MIDAS software, a microarray data analysis system (http://www.tm4.org/midas.html) using the LOWESS (Localized Weighted Smother Estimator) method. The raw microarray data have been submitted to the MIAME ArrayExpress database (miamexpress@ebi.ac.uk) with accession number E-MEXP-2949.

The normalized data were analyzed in order to select a discrete number of differentially regulated genes with a threshold level for False Discovery Rate (FDR) <1%. Normalized expression values were used as input for the Significance Analysis of Microarray (SAM) software (http://www-stat.stanford.edu/~tibs/SAM/). This software assigns a score to each gene based on the change in gene expression relative to the standard deviation of repeated measurements. For genes with scores greater than an adjustable threshold (delta), SAM uses permutations of the repeated measurements to estimate the percentage of genes identified by chance, the FDR. Setting the delta value at 1.212, the FDR of the selected genes was equal to zero and after removing genes represented in the array by more than one spot and false genes SAM extracts significantly differentially expressed genes. Expression value ratios (S/N and N/C) between the two channels were then transformed to logarithmic scale base 2 (log_2_ ratio). Then data analysis was the done using the KEGG database to find genes affected in well known pathways.

### GST pull-down assay and *in vitro* binding

GST-tagged recombinant proteins were expressed as detailed in a previous paper [15]. Lysates were prepared from transfected COS7 cells expressing PML or YB-1, and from transfected U2OS cells expressing telethonin/Tcap. Untransfected COS7 cells were used as a source of endogenous p53. Equal amounts of GST fusion proteins immobilized on glutathione-Sepharose beads were incubated from 2–12 hours at 4°C with cell lysate in binding buffer: 50 mM HEPES (pH 7.0), 250 mM NaCl, 0.1% NP-40 and protease inhibitors (Roche). Immobilized protein complexes were washed with binding buffer and separated by SDS-PAGE. A plasmid coding for human wild type ZO-1 was used as the template for an IVTT reaction in the presence of [^35^S] methionine producing radiolabeled ZO-1. This protein was used in *in vitro* binding assays with GST-Ankrd2 protein bound to glutathione-Sepharose 4B or GST alone, incubated for three hours at RT, washed and then subjected to SDS-PAGE.

### Immunoblotting

Protein complexes, resolved by SDS PAGE, were transferred to PVDF membrane (Immobilon P, Millipore) as previously reported [14]. Proteins were visualized using the ECL chemiluminescence detection system (Millipore). The primary antibodies anti-p53 (DO-1, Santa Cruz), anti-FLAG (M2, Sigma), anti-telethonin/Tcap, anti-p50 (Santa Cruz) and anti H3 (Santa Cruz), as well as secondary anti-mouse and anti-rabbit antibodies conjugated with horseradish peroxidase (Sigma and Pierce, respectively) were used for detection of p53, YB-1, PML, telethonin/Tcap, NFκB subunit p50 and histone H3.

### Protein arrays

PDZ and SH3 array membranes (Panomics/Affymetrix, USA) were used according to the protocols in the manufacturer's handbook. His-tagged Ankrd2 protein (15 µg/ml) was used as a ligand. The protein-protein and protein-DNA interaction assays were carried out using the TransSignal Transcription Factor (TF) Protein Array II (Panomics/Affymetrix, USA) according to the manufacturer's instructions. Briefly, purified Ankrd2-GST protein or a DNA probe containing the *Ankrd2* promoter region (−1173 to −4 bp) amplified by PCR using biotinylated primers, were incubated with TF protein array membranes. The interactions were detected either using mouse anti-GST antibody and then HRP-conjugated goat anti-mouse antibody (Sigma) to detect GST-Ankrd2 bound to the spotted proteins on the membranes, or streptavidin-HRP antibody, to detect the biotinylated DNA probe. The signals were visualized by chemiluminescence.

### Luciferase Assays

SaOs2 and C2C12 cells were grown for 24 h and then transiently co-transfected with the reporter construct Ankrd2 (−439/+7)-LUC, expression vectors for p53, Nkx2.5, Ankrd1/CARP or MyoD, and a control plasmid expressing *Renilla* luciferase, pRL-TK (Promega). The total amount of DNA was kept constant by the addition of empty vector; pCDNA3. In order to activate the NFkB transcription factor, cells were incubated with 0.1, 1 and 20 ng/ml of TNF α (Promega) for 20 h. The cells were lysed in Passive Lysis Buffer (Promega) and luciferase activity was measured using the Dual Luciferase Reporter Assay System (Promega) according to the manufacturer's instructions. The firefly luciferase activity was normalized against *Renilla* luciferase and the means of three independent experiments performed in triplicate were calculated. Data were presented as means ± standard error of the mean. Individual means were compared using the Student t-test. Differences were considered to be statistically significant at p<0.05.

## Supporting Information

Figure S1 **Silencing of endogenous Ankrd2 in differentiated human skeletal muscle cells, using an AdenoAssociated Virus (AAV) vector AAV-shRNAex1-2.** The sequence of the siRNA from *Ankrd2* exon 1–2 that reduced the expression of exogenous Ankrd2 in transfected cells was used to design both sense and antisense oligonucleotides (21 nucleotides). These were annealed and cloned as a ds oligo into the pZAC−U6−CMV−ZsGreen plasmid that contains a U6 promoter for RNA polymerase III transcription of shRNA and a CMV promoter for expression of the ZsGreen fluorescent protein. In order to have a good level of endogenous Ankrd2 expression for silencing AAV-shRNAex1-2 was used to infect already differentiating (after 5 days differentiation) primary human muscle cells (CHQ5B). Cells were also infected with AAV-shLuc (N) as a negative control. Cells were harvested 4 days after infection (total of 9 days differentiation). Total RNA was extracted and analyzed by RT-PCR for Ankrd2 and GAPDH expression (two upper panels). The cell lysates were analyzed by Western blot for expression of Ankrd2, ZsGreen, Myosin Heavy Chain (MHC) and GAPDH (lower four panels). GAPDH was used as a loading control, MHC as an indicator of differentiation and ZsGreen as a control of expression of AAV-shRNAex1-2. In cells infected with AAV-shRNAex1-2 (S) the endogenous Ankrd2 is significantly reduced both at the RNA and protein level compared to its levels in non-silenced cells infected with AAV-shLuc (N) and uninfected control cells (C).
(TIF)

Figure S2 **Coomassie blue stained gels demonstrating equal amounts of purified GST, GST tagged Ankrd2 and its deletants separated by SDS-PAGE.** The same amounts of proteins were used in GST pull down reactions in mapping experiments, panel A corresponds to Figure 1B and panel B to Figure 1C. Purified recombinant proteins are designated as: **A**, almost full length Ankrd2 protein (aa 5–333); **sA**, Ankrd2 protein with a 97 aa Nterminal deletion (aa 98–333); **N**, N-terminal (aa 5–120); **NA**, N-terminal plus ankyrin repeats (aa 5–284); **CA**, C-terminal plus ankyrin repeats (aa 121–333); **C**, C-terminal (aa 280–333). Molecular size of proteins is given on the left, in kDa.
(TIF)

Table S1 **Genes downregulated in Ankrd2 silenced myotubes.**
(DOC)

Table S2 **Genes upregulated in Ankrd2 silenced myotubes.**
(DOC)

Table S3 **Differentially expressed genes in infected (non silenced) compared to uninfected CHQ5B cells.**
(DOC)

Table S4 **KEGG pathways with 7 or more genes differentially expressed in Ankrd2 silenced myotubes.**
(DOC)
