# Supplementary figures and images for: Multi-Tasking Role of the Mechanosensing Protein Ankrd2 in the Signaling Network of Striated Muscle
Source: PLoS One. 2011 Oct 10;6(10):e25519. doi: 10.1371/journal.pone.0025519 (PMC3189947; doi:10.1371/journal.pone.0025519)

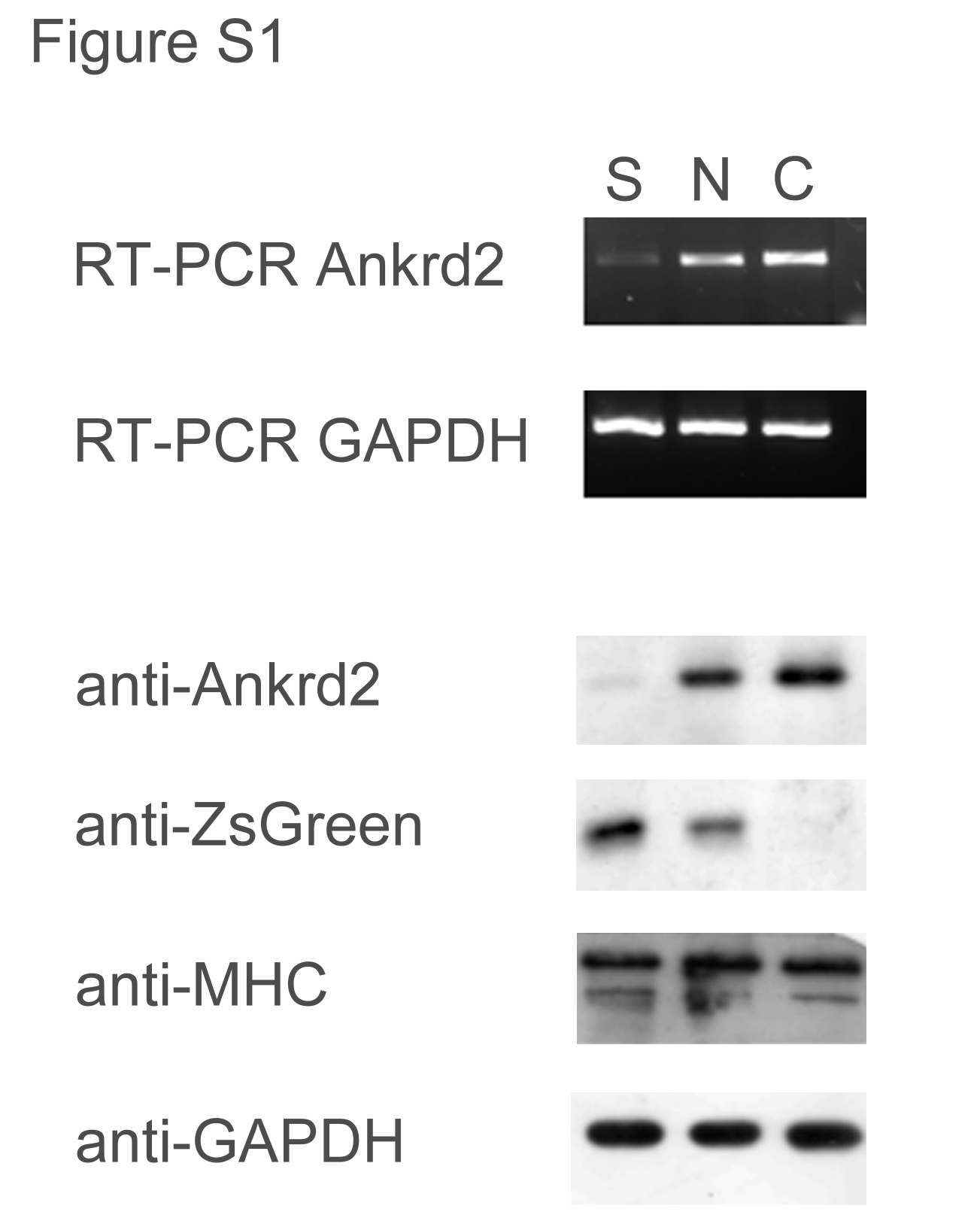

Supplement: Figure S1 — Silencing of endogenous Ankrd2 in differentiated human skeletal muscle cells, using an AdenoAssociated Virus (AAV) vector AAV-shRNAex1-2. The sequence of the siRNA from Ankrd2 exon 1–2 that reduced the expression of exogenous Ankrd2 in transfected cells was used to design both sense and antisense oligonucleotides (21 nucleotides). These were annealed and cloned as a ds oligo into the pZAC−U6−CMV−ZsGreen plasmid that contains a U6 promoter for RNA polymerase III transcription of shRNA and a CMV promoter for expression of the ZsGreen fluorescent protein. In order to have a good level of endogenous Ankrd2 expression for silencing AAV-shRNAex1-2 was used to infect already differentiating (after 5 days differentiation) primary human muscle cells (CHQ5B). Cells were also infected with AAV-shLuc (N) as a negative control. Cells were harvested 4 days after infection (total of 9 days differentiation). Total RNA was extracted and analyzed by RT-PCR for Ankrd2 and GAPDH expression (two upper panels). The cell lysates were analyzed by Western blot for expression of Ankrd2, ZsGreen, Myosin Heavy Chain (MHC) and GAPDH (lower four panels). GAPDH was used as a loading control, MHC as an indicator of differentiation and ZsGreen as a control of expression of AAV-shRNAex1-2. In cells infected with AAV-shRNAex1-2 (S) the endogenous Ankrd2 is significantly reduced both at the RNA and protein level compared to its levels in non-silenced cells infected with AAV-shLuc (N) and uninfected control cells (C). (TIF) [file pone.0025519.s001.tif]
