# Supplementary material for: Multi-Tasking Role of the Mechanosensing Protein Ankrd2 in the Signaling Network of Striated Muscle
Source: PLoS One. 2011 Oct 10;6(10):e25519. doi: 10.1371/journal.pone.0025519 (PMC3189947; doi:10.1371/journal.pone.0025519)
Supplement: Table S1 — Genes downregulated in Ankrd2 silenced myotubes. (DOC) [file pone.0025519.s003.doc]

**Table S1. Genes downregulated in Ankrd2 silenced myotubes**

| **Gene Symbol** | **Log2 ratio** | **Gene Description** |
| --- | --- | --- |
| MYBPC1 | -2.77 | myosin binding protein C, slow type |
| ANKRD2 | -2.02 | ankyrin repeat domain 2 (stretch responsive muscle) |
| E2F8 | -2.00 | E2F transcription factor 8 |
| AMPD1 | -1.95 | adenosine monophosphate deaminase 1 (isoform M) |
| APOBEC2 | -1.95 | apolipoprotein B mRNA editing enzyme, |
| NBR1 | -1.83 | neighbor of BRCA1 gene |
| PHKG1 | -1.74 | phosphorylase kinase, gamma 1 (muscle), AA495894 |
| ART3 | -1.73 | ADP-ribosyltransferase 3 |
| DEPDC6 | -1.72 | DEP domain containing 6 |
| AGL | -1.67 | amylo-1, 6-glucosidase |
| PPARGC1A | -1.61 | peroxisome proliferator-activated receptor |
| CGNL1 | -1.61 | cingulin-like 1 |
| TXLNB | -1.60 | taxilin beta |
| SLN | -1.58 | sarcolipin |
| FAM78A | -1.56 | family with sequence similarity 78, member A |
| GREM2 | -1.52 | gremlin 2, cysteine knot superfamily, homolog |
| ATP2A1 | -1.51 | ATPase, Ca transporting, cardiac muscle, |
| PEG10 | -1.51 | paternally expressed 10 |
| CRB1 | -1.50 | crumbs homolog 1 (Drosophila) |
| ZWINT | -1.50 | ZW10 interactor |
| PDK4 | -1.49 | pyruvate dehydrogenase kinase, isozyme 4 |
| AGPAT5 | -1.48 | 1-acylglycerol-3-phosphate O-acyltransferase 5 |
| MYCL1 | -1.47 | v-myc myelocytomatosis viral oncogene homolog 1 |
| CAPN6 | -1.46 | calpain 6 |
| MIB1 | -1.45 | mindbomb homolog 1 (Drosophila), AK123912 |
| CMYA3 | -1.44 | cardiomyopathy associated 3 |
| ZNF426 | -1.43 | zinc finger protein 426 |
| TMEM158 | -1.42 | transmembrane protein 158 |
| PLEKHO1 | -1.42 | pleckstrin homology domain family O |
| EGLN3 | -1.41 | egl nine homolog 3 (C. elegans) |
| ITIH4 | -1.41 | inter-alpha (globulin) inhibitor H4 |
| CTXN3 | -1.40 | cortexin 3 |
| NASP | -1.39 | nuclear autoantigenic sperm protein |
| ABCA1 | -1.39 | ATP-binding cassette, sub-family A 1 |
| TOMM40L | -1.39 | translocase of outer mitochondrial membrane |
| MYOZ1 | -1.39 | myozenin 1/FATZ-1/calsarcin2 |
| TRIM45 | -1.38 | tripartite motif-containing 45 |
| IGFN1 | -1.37 | eEF1A2 binding protein 1 |
| C16orf53 | -1.36 | chromosome 16 open reading frame 53 |
| LRRC39 | -1.35 | leucine rich repeat containing 39 |
| LOC92196 | -1.34 | similar to death-associated protein |
| KIAA1217 | -1.34 | sickle tail protein homolog |
| CSRP3 | -1.32 | cysteine and glycine-rich protein 3 |
| PPP1R12B | -1.31 | protein phosphatase 1, subunit 12B |
| ANP32A | -1.27 | acidic nuclear phosphoprotein 32 family |
| C6orf142 | -1.26 | chromosome 6 open reading frame 142 |
| ITGB8 | -1.26 | integrin, beta 8 |
| MLLT3 | -1.24 | myeloid/lymphoid or mixed-lineage leukemia |
| LGI1 | -1.23 | leucine-rich, glioma inactivated 1 |
| DNAJC12 | -1.23 | DnaJ homolog, subfamily C, member 12 |
| GCNT3 | -1.23 | glucosaminyl (N-acetyl) transferase 3 |
| TMEM9 | -1.23 | transmembrane protein 9 |
| PHKG1 | -1.22 | phosphorylase kinase, gamma 1 (muscle) |
| LRP1B | -1.21 | low density lipoprotein-related protein 1B |
| THRSP | -1.21 | thyroid hormone responsive |
| MYOT | -1.20 | myotilin |
| SLC24A3 | -1.20 | solute carrier family 24 (Na/K/Ca exchanger) |
| TMEM14B | -1.19 | transmembrane protein 14B |
| CABC1 | -1.18 | chaperone, ABC1 activity of bc1 complex |
| LRTM1 | -1.18 | leucine-rich repeats/ transmembrane domain1 |
| LIN9 | -1.18 | lin-9 homolog (C. elegans) |
| PIK3C2B | -1.18 | phosphoinositide-3-kinase, class 2 beta |
| TNNT3 | -1.17 | troponin T type 3 (skeletal, fast) |
| MYOM1 | -1.17 | myomesin 1 (skelemin) 185kDa |
| PPT1 | -1.17 | palmitoyl-protein thioesterase 1 |
| GOLPH3L | -1.17 | golgi phosphoprotein 3-like |
| TJP2 | -1.16 | tight junction protein 2 (zona occludens) |
| ENGASE | -1.15 | Endo-beta-N-acetylglucosaminidase, LOC92196 |
| FRAT2 | -1.15 | rearranged in advanced T-cell lymphomas 2 |
| PYGM | -1.15 | phosphorylase, glycogen; muscle |
| FZD4 | -1.14 | frizzled homolog 4 (Drosophila) |
| TIFA | -1.14 | TRAF-interacting protein |
| HADH | -1.14 | Coenzyme A dehydrogenase |
| MKRN1 | -1.14 | makorin, ring finger protein, 1 |
| SLC9A9 | -1.14 | solute carrier family 9 |
| SNRPB2 | -1.13 | small nuclear ribonucleoprotein polypeptide B |
| STK31 | -1.13 | serine/threonine kinase 31 |
| KIAA1191 | -1.12 | Brain-derived rescue factor p60 MONOX |
| KRR1 | -1.12 | KRR1, small subunit processome component |
| DPY19L1 | -1.12 | dpy-19-like 1 (C. elegans) |
| TEX14 | -1.12 | testis expressed sequence 14 |
| RPS17 | -1.11 | ribosomal protein S17 |
| TNNC2 | -1.11 | troponin C type 2 (fast) |
| SSPN | -1.11 | sarcospan (Kras oncogene-associated gene) |
| HOP | -1.10 | homeodomain-only protein |
| XK | -1.10 | X-linked Kx blood group (McLeod syndrome) |
| DUSP26 | -1.10 | dual specificity phosphatase 26 (putative) |
| CLGN | -1.10 | calmegin |
| TMTC4 | -1.09 | transmembrane and tetratricopeptide repeat |
| ATG16L2 | -1.08 | ATG16 autophagy related 16-like 2 |
| HSPD1 | -1.08 | heat shock 60kDa protein 1 (chaperonin) |
| MEF2C | -1.08 | MADS box transcription enhancer factor 2 |
| PITX2 | -1.07 | paired-like homeodomain TF 2 |
| TOMM20 | -1.07 | translocase of outer mitochondrial membrane |
| PDE4DIP | -1.07 | phosphodiesterase 4D interacting protein |
| SSTR1 | -1.06 | somatostatin receptor 1 |
| ALPK1 | -1.05 | alpha-kinase 1 |
| LACTB2 | -1.05 | lactamase, beta 2 |
| ZNF533 | -1.05 | zinc finger protein 533 |
| RGS2 | -1.05 | regulator of G-protein signalling 2, 24kDa |
| FAM13A1 | -1.05 | family with sequence similarity 13, member A1 |
| SMARCE1 | -1.05 | SWI/SNF related, matrix associated |
| NOLC1 | -1.04 | nucleolar and coiled-body phosphoprotein 1 |
| TRAM1L1 | -1.04 | translocation assoc. membrane protein 1-like |
| GFM2 | -1.04 | G elongation factor, mitochondrial 2 |
| CYCS | -1.04 | cytochrome c, somatic |
| SCARNA15 | -1.03 | small Cajal body-specific RNA 15, BX538250 |
| SMN1 | -1.03 | survival of motor neuron 1, telomeric |
| ECM2 | -1.03 | extracellular matrix protein 2, |
| TTN | -1.03 | titin |
| ZNF721 | -1.03 | zinc finger protein 721 |
| ZBED3 | -1.02 | zinc finger, BED-type containing 3 |
| RRAGD | -1.02 | Ras-related GTP binding D |
| METTL9 | -1.02 | methyltransferase like 9 |
| CUL7 | -1.02 | cullin 7 |
| CSRP2 | -1.02 | cysteine and glycine-rich protein 2 |
| MYLIP | -1.02 | myosin regulatory LC interacting protein |
| MLYCD | -1.01 | malonyl-CoA decarboxylase |
| PLCL2 | -1.01 | phospholipase C-like 2 |
| MMP7 | -1.01 | matrix metallopeptidase 7 (matrilysin, uterine) |
| CSRP2BP | -1.01 | CSRP2 binding protein |
| TUBA8 | -1.00 | tubulin, alpha 8 |
| KHNYN | -1.00 | KH and NYN domain containing protein, KIAA0323 |
| CCDC25 | -1.00 | coiled-coil domain containing 25 protein |
| METTL7A | -0.99 | methyltransferase like 7A |
| AKR1B10 | -0.99 | aldo-keto reductase family 1, member B10 (aldose reductase) |
| KLHL3 | -0.99 | kelch-like 3 (Drosophila) |
| BCAT1 | -0.99 | branched chain aminotransferase 1, cytosolic |
| IL17B | -0.99 | interleukin 17B |
| HES1 | -0.99 | hairy and enhancer of split 1, (Drosophila) |
| ZNF727 | -0.99 | zinc finger protein 727 (LOC442319) |
| ADSSL1 | -0.98 | adenylosuccinate synthase like 1 |
| SMYD1 | -0.98 | SET and MYND domain containing |
| PDLIM3 | -0.98 | ALP, PDZ and LIM domain3 |
| SYTL3 | -0.98 | synaptotagmin-like 3 |
| MYL1 | -0.98 | myosin light chain 1, skeletal, fast |
| CORO6 | -0.98 | coronin 6 |
| FAM49B | -0.97 | family with sequence similarity 49, member B |
| ST8SIA5 | -0.97 | ST8 alpha-N-acetyl-neuraminide alpha-2,8-sialyltransferase 5 |
| MYF6 | -0.97 | myogenic factor 6 (herculin) |
| SACM1L | -0.97 | SAC1 suppressor of actin mutations 1-like (yeast) |
| NEXN | -0.97 | nexilin (F actin binding protein) |
| C1orf213 | -0.97 | chromosome 1 open reading frame 213 |
| CEP350 | -0.96 | centrosomal protein 350kDa |
| ZNF493 | -0.96 | zinc finger protein 493 |
| C17orf86 | -0.96 | chromosome 17 open reading frame 86 |
| DDIT4L | -0.96 | DNA-damage-inducible transcript 4-like REED2 |
| BHLHB3 | -0.96 | basic helix-loop-helix family, member e41 |
| SERPINB6 | -0.95 | serpin peptidase inhibitor, clade B (ovalbumin), member 6 |
| IFIT1 | -0.95 | Interferon-induced 56 kDa protein |
| TM7SF3 | -0.94 | transmembrane 7 superfamily member 3 |
| NEAT1 | -0.94 | nuclear paraspeckle assembly transcript 1 (non-protein coding) |
| ABLIM2 | -0.94 | actin binding LIM protein family, member 2 |
| ZNF254 | -0.94 | zinc finger protein 254 |
| FOXO3A | -0.94 | FOXO3 forkhead box O3 |
| POPDC2 | -0.94 | popeye domain containing 2 |
| SCARA5 | -0.93 | scavenger receptor class A, member 5 (putative) |
| BCL6 | -0.93 | B-cell CLL/lymphoma 6 |
| LUC7L3 | -0.93 | LUC7-like 3 (S. cerevisiae), CROP |
| ANK3 | -0.93 | ankyrin 3, node of Ranvier (ankyrin G) |
| c2orf69 | -0.92 | chromosome 2 open reading frame 69, FLJ38973 |
| GTF2F1 | -0.92 | general transcription factor IIF, polypeptide |
| NPM1 | -0.92 | nucleophosmin (nucleolar phosphoprotein B23, numatrin) |
| PTMA | -0.92 | prothymosin, alpha |
| ZNF680 | -0.92 | zinc finger protein 680 |
| RNF150 | -0.92 | ring finger protein 150 |
| VTI1B | -0.92 | vesicle transport through interaction with t-SNAREs homolog 1B |
| ZNF714 | -0.92 | zinc finger protein 714 |
| BTG3 | -0.92 | BTG family, member 3 |
| LRRN1 | -0.92 | leucine rich repeat neuronal 1 |
| PFKM | -0.92 | phosphofructokinase, muscle |
| FAM173B | -0.92 | family with sequence similarity 1,73 member B, LOC134145 |
| KAT2A | -0.92 | K(lysine) acetyltransferase 2A, GCN5L2 |
| NEFH | -0.91 | neurofilament, heavy polypeptide |
| LOC100128178 | -0.91 | similar to hCG2041313, AK021543 |
| MRPS36 | -0.91 | mitochondrial ribosomal protein S36 |
| SEMA3D | -0.91 | sema domain, immunoglobulin domain (Ig) |
| CCDC90B | -0.91 | coiled-coil domain containing 90B |
| TMEM100 | -0.91 | transmembrane protein 100 |
| KGFLP1 | -0.91 | keratinocyte growth factor-like protein 1 |
| MRPS25 | -0.91 | mitochondrial ribosomal protein S25 |
| SMARCA5 | -0.91 | SWI/SNF related, actin dependent regulator of chromatin |
| SYNPO2L | -0.91 | synaptopodin 2-like |
| RPS6KA5 | -0.91 | ribosomal protein S6 kinase, 90kDa, polypeptide 5 |
| ARNT | -0.90 | aryl hydrocarbon receptor nuclear translocator |
| CX40.1 | -0.90 | gap junction protein, delta 4, 40.1kD, Connexin-40.1 |
| FLJ37453 | -0.90 | hypothetical LOC729614 |
| HERC2 | -0.90 | hect domain and RLD 2, jdf2 |
| ADCY3 | -0.90 | adenylate cyclase 3 |
| ZNF783 | -0.90 | zinc finger family member 783 |
| IMPACT | -0.90 | Impact homolog (mouse) |
| LOC152217 | -0.90 | hypothetical LOC152217 |
| IFI44L | -0.89 | interferon-induced protein 44-like |
| DENND5B | -0.89 | DENN/MADD domain containing 5, MGC24039 |
| MYL5 | -0.89 | myosin, light chain 5, regulatory |
| DAAM1 | -0.89 | dishevelled associated activator of morphogenesis 1 |
| PPM1E | -0.89 | protein phosphatase, Mg2+/Mn2+ dependent, 1E |
| MGST1 | -0.89 | microsomal glutathione S-transferase 1 |
| HMG4L | -0.89 | HMGB3L, high-mobility group box 3-like 1 |
| PDCD7 | -0.89 | programmed cell death 7 |
| ABCA5 | -0.88 | ATP-binding cassette, sub-family A , member 5 |
| DKC1 | -0.88 | dyskeratosis congenita 1, dyskerin |
| HSDL2 | -0.88 | hydroxysteroid dehydrogenase like 2 |
| FAM184A | -0.88 | family with sequence similarity 184, C6orf60 |
| ACAN (AGC1) | -0.88 | aggrecan |
| PPP1R9A | -0.87 | protein phosphatase 1, regulatory (inhibitor) subunit 9A |
| HIST1H2AC | -0.87 | histone cluster 1, H2ac |
| CP110 | -0.87 | CP110 protein |
| TBX15 | -0.87 | T-box transcription factor 15 |
| C20orf166 | -0.87 | chromosome 20 open reading frame 166 |
| UBE2D1 | -0.87 | ubiquitin-conjugating enzyme E2D 1 (UBC4/5 homolog, yeast) |
| MYPN | -0.87 | myopalladin |
| PDLIM5 | -0.87 | PDZ and LIM domain 5, Enigma-like protein |
| C17orf79 | -0.86 | chromosome 17 open reading frame 79 |
| ENPP5 | -0.86 | ectonucleotide pyrophosphatase/phosphodiesterase 5 |
| PTGER2 | -0.86 | prostaglandin E receptor 2 (subtype EP2), 53kDa |
| FLJ10038 | -0.86 | hypothetical protein FLJ10038 |
| CENPW | -0.86 | centromere protein W centromere protein W, C6orf173 |
| APOC1 | -0.86 | apolipoprotein C-I |
| ZNF555 | -0.86 | zinc finger protein 555 |
| SPTLC3 | -0.86 | serine palmitoyltransferase, |
| FEZ1 | -0.86 | fasciculation and elongation protein zeta 1 (zygin I) |
| AMY1C | -0.86 | amylase, alpha 1A (salivary) |
| FAM53B | -0.86 | family with sequence similarity 53, member B |
| PIK3IP1 | -0.85 | phosphoinositide-3-kinase interacting protein 1 (MGC17330) |
| CNIH3 | -0.85 | cornichon homolog 3 (Drosophila) |
| C20orf177 | -0.85 | chromosome 20 open reading frame 177 |
| UNC45B | -0.85 | unc-45 homolog B (C. elegans) |
| HIRA | -0.85 | HIR histone cell cycle regulation defective homolog A |
| DNAJC18 | -0.85 | dnaJ homolog subfamily C member 18 |
| LOC643837 | -0.85 | hypothetical LOC643837 |
| EID1 | -0.85 | EP300 interacting inhibitor of differentiation 1 |
| ACVR1 | -0.85 | activin A receptor, type I, Ser/thr-protein kinase receptor R1 |
| MAMLD1 | -0.85 | mastermind-like domain containing 1, CXorf6 |
| GPD1L | -0.85 | glycerol-3-phosphate dehydrogenase 1-like |
| YIPF7 | -0.85 | Yip1 domain family, member 7 |
| ZNF227 | -0.84 | zinc finger protein 227 |
| PLCD4 | -0.84 | phospholipase C, delta 4 |
| CLEC2D | -0.84 | C-type lectin domain family 2, member D |
| MRPL23 | -0.84 | mitochondrial ribosomal protein L23 |
| C8orf40 | -0.84 | chromosome 8 open reading frame 40 |
| LOC100128239 | -0.84 | hypothetical LOC100128239, AK125302 |
| FLJ22536 | -0.84 | hypothetical locus LOC401237 |
| RAD23B | -0.84 | RAD23 homolog B (S. cerevisiae) |
| IFI44 | -0.84 | interferon-induced protein 44 |
| NRIP1 | -0.83 | nuclear receptor interacting protein 1 |
| CYP1B1 | -0.83 | cytochrome P450, family 1, subfamily B, polypeptide 1 |
| FDX1 | -0.83 | ferredoxin 1 |
| HCFC1R1 | -0.83 | host cell factor C1 regulator 1 (XPO1 dependent) |
| KIAA0146 | -0.83 | KIAA0146 |
| ARHGEF37 | -0.83 | Rho guanine nucleotide exchange factor (GEF) 37, FLJ41603 |
| SIX4 | -0.83 | SIX homeobox 4 |
| CKM | -0.83 | creatine kinase, muscle |
| GPNMB | -0.83 | glycoprotein (transmembrane) nmb |
| MFSD1 | -0.83 | major facilitator superfamily domain containing 1 |
| FLJ34077 | -0.83 | weakly similar to zinc finger protein 195 |
| MYH8 | -0.83 | myosin, heavy chain 8, skeletal muscle, perinatal |
| TNNI2 | -0.83 | troponin I type 2 (skeletal, fast) |
| AKAP2 | -0.82 | A kinase (PRKA) anchor protein 2 |
| GLUL | -0.82 | glutamate-ammonia ligase (glutamine synthetase) |
| BEX2 | -0.82 | brain expressed X-linked 2 |
| RPS6KB1 | -0.82 | ribosomal protein S6 kinase, 70kDa, polypeptide 1 |
| HEY1 | -0.82 | hairy/enhancer-of-split related with YRPW motif 1 |
| C1orf9 | -0.82 | chromosome 1 open reading frame 9 |
| CYP27A1 | -0.82 | cytochrome P450, family 27, subfamily A, polypeptide 1 |
| IL6R | -0.81 | interleukin 6 receptor |
| HSF2 | -0.81 | heat shock transcription factor 2 |
| ATP6V1E2 | -0.81 | ATPase, H+ transporting, lysosomal 31kDa, V1 subunit E2 |
| LAMA4 | -0.81 | laminin, alpha 4 |
| BTN3A2 | -0.81 | butyrophilin, subfamily 3, member A2 |
| UBE1C | -0.81 | ubiquitin-like modifier activating enzyme 3 |
| EGR2 | -0.81 | early growth response 2 |
| VASR2 | -0.81 | valyl-tRNA synthetase 2, mitochondrial (putative) |
| CIT | -0.81 | citron (rho-interacting, serine/threonine kinase 21) |
| ZNF91 | -0.81 | zinc finger protein 91 |
| DNALI1 | -0.81 | dynein, axonemal, light intermediate chain 1 |
| AARSD1 | -0.80 | alanyl-tRNA synthetase domain containing 1 |
| LRIG1 | -0.80 | leucine-rich repeats and immunoglobulin-like domains 1 |
| BRD3 | -0.80 | bromodomain containing 3 |
| TRIT1 | -0.80 | tRNA isopentenyltransferase 1 |
| UTP3 | -0.80 | small subunit (SSU) processome component, homolog , SAS10 |
| C21orf62 | -0.80 | chromosome 21 open reading frame 62 |
