# Supplementary material for: Multi-Tasking Role of the Mechanosensing Protein Ankrd2 in the Signaling Network of Striated Muscle
Source: PLoS One. 2011 Oct 10;6(10):e25519. doi: 10.1371/journal.pone.0025519 (PMC3189947; doi:10.1371/journal.pone.0025519)
Supplement: Table S2 — Genes upregulated in Ankrd2 silenced myotubes. (DOC) [file pone.0025519.s004.doc]

**Table S2. Genes upregulated in Ankrd2 silenced myotubes**

| **Gene Symbol** | **Log2 ratio** | **Gene Description** |
| --- | --- | --- |
| DEFB103A | 3.53 | defensin, beta 103A |
| PSG1 | 3.49 | pregnancy specific beta-1-glycoprotein 1 |
| PSG6 | 3.05 | pregnancy specific beta-1-glycoprotein 6 |
| KCNE1L | 2.99 | KCNE1-like |
| PSG8 | 2.87 | pregnancy specific beta-1-glycoprotein 8 |
| D4S234 | 2.87 | DNA segment on chromosome 4 (unique) |
| KISS1 | 2.70 | KiSS-1 metastasis-suppressor |
| AES | 2.43 | amino-terminal enhancer of split, AF086187 |
| MAL | 2.42 | mal, T-cell differentiation protein |
| GSG1 | 2.38 | germ cell associated 1 |
| KRT34 | 2.29 | keratin 34 |
| SFRP4 | 2.24 | secreted frizzled-related protein 4 |
| FOXJ1 | 2.19 | forkhead box J1 |
| NPAS1 | 2.17 | neuronal PAS domain protein 1 |
| PSG2 | 2.15 | pregnancy specific beta-1-glycoprotein 2 |
| HTR3E | 2.11 | 5-hydroxytryptamine (serotonin) receptor 3 |
| GRASP | 2.10 | GRP1 (general receptor for phosphoinositides 1) |
| PSG7 | 2.03 | pregnancy specific beta-1-glycoprotein 7 |
| AQP1 | 2.01 | aquaporin 1 (Colton blood group) |
| GNAZ | 2.00 | guanine nucleotide binding protein (G protein) |
| CRIP1 | 1.94 | cysteine-rich protein 1 (intestinal) |
| BBX | 1.94 | bobby sox homolog (Drosophila), BC037919 |
| MMP11 | 1.92 | matrix metallopeptidase 11 (stromelysin 3) |
| CEP78 | 1.90 | centrosomal protein 78kDa |
| NGFR | 1.88 | nerve growth factor receptor (TNFR superfamily) |
| RAB15 | 1.87 | RAB15, member RAS onocogene family |
| SCT | 1.84 | secretin |
| BCAN | 1.81 | brevican |
| STMN2 | 1.79 | stathmin-like 2 |
| PDGFRB | 1.77 | platelet-derived growth factor receptor |
| KREMEN2 | 1.77 | kringle containing transmembrane protein 2 |
| CTSH | 1.77 | cathepsin H |
| GPR123 | 1.73 | G protein-coupled receptor 123 |
| TRIM59 | 1.72 | tripartite motif-containing 59 |
| BPY2B | 1.71 | basic charge, Y-linked, 2B; BQ130701 |
| EDN1 | 1.70 | endothelin 1 |
| PLEKHG5 | 1.69 | pleckstrin homology domain family, 5 |
| TNFRSF25 | 1.69 | tumor necrosis factor receptor superfamily, 25 |
| FARP1 | 1.67 | FERM, RhoGEF (ARHGEF) and pleckstrin domain |
| AMIGO2 | 1.65 | adhesion molecule with Ig-like domain 2 |
| ITGA6 | 1.64 | integrin, alpha 6 |
| ATP10A | 1.64 | ATPase, Class V, type 10A |
| EML1 | 1.63 | echinoderm microtubule associated protein like 1 |
| SPRR2G | 1.61 | small proline-rich protein 2G |
| CR2 | 1.60 | complement component (3d/E B virus) receptor 2 (CR740121) |
| PSG9 | 1.60 | pregnancy specific beta-1-glycoprotein 9 |
| VASH1 | 1.60 | vasohibin 1 |
| PSG11 | 1.53 | pregnancy specific beta-1-glycoprotein 11 |
| UHMK1 | 1.52 | U2AF homology motif (UHM) kinase 1 |
| PALM | 1.48 | paralemmin |
| GPRC5C | 1.47 | G protein-coupled receptor, family C, group 5, |
| ATP8B2 | 1.45 | ATPase, Class I, type 8B, member 2 |
| FSTL3 | 1.44 | follistatin-like 3 (secreted glycoprotein) |
| TGFB2 | 1.41 | transforming growth factor, beta 2 |
| CAV1 | 1.41 | caveolin 1, caveolae protein, 22kDa |
| RASSF7 | 1.41 | Ras association domain family (N-terminal) member 7 |
| COL19A1 | 1.38 | collagen, type XIX, alpha 1 |
| KRT31 | 1.40 | keratin 31 |
| MYOZ2 | 1.39 | myozenin 2/FATZ-2/calsarcin-1 |
| FOLR1 | 1.39 | folate receptor 1 (adult) |
| YES1 | 1.39 | Yamaguchi sarcoma viral oncogene homolog 1 |
| SOD3 | 1.39 | superoxide dismutase 3, extracellular |
| KHK | 1.38 | ketohexokinase (fructokinase) |
| COL20A1 | 1.38 | collagen, type XX, alpha 1 |
| F10 | 1.38 | coagulation factor X |
| MAP2K1 | 1.38 | mitogen-activated protein kinase kinase 1 |
| EFNA1 | 1.38 | ephrin-A1 |
| EPHA4 | 1.37 | EPH receptor A4 |
| OTUD1 | 1.35 | OTU domain containing 1 |
| ELF4 | 1.33 | E74-like factor 4 (ets domain transcription factor) |
| TAS1R1 | 1.32 | taste receptor, type 1, member 1 |
| ISOC1 | 1.32 | isochorismatase domain containing 1 |
| PCNP | 1.32 | PEST proteolytic signal containing nuclear protein |
| TSPYL4 | 1.31 | TSPY-like 4 |
| VDR | 1.30 | vitamin D (1,25- dihydroxyvitamin D3) receptor |
| HBEGF | 1.30 | heparin-binding EGF-like growth factor |
| RPESP | 1.30 | RPE-spondin |
| MAGT1 | 1.29 | implantation-associated protein |
| RASSF2 | 1.28 | Ras association (RalGDS/AF-6) domain family 2 |
| NANOS1 | 1.27 | nanos homolog 1 (Drosophila) |
| PLCB4 | 1.26 | phospholipase C, beta 4 |
| PRKAG2 | 1.26 | protein kinase, AMP-activated, gamma |
| SERPINE1 | 1.26 | serpin peptidase inhibitor, clade E (nexin) |
| ADRA1B | 1.25 | adrenergic, alpha-1B-, receptor |
| MTMR2 | 1.24 | myotubularin related protein 2 |
| KRT80 | 1.23 | keratin 80 |
| SC4MOL | 1.23 | sterol-C4-methyl oxidase-like |
| FILIP1L | 1.23 | filamin A interacting protein 1-like |
| AK1 | 1.23 | adenylate kinase 1, AK126976 |
| PTPRF | 1.22 | protein tyrosine phosphatase, receptor type, F |
| RAB3IP | 1.22 | RAB3A interacting protein (rabin3) |
| MARCH4 | 1.22 | membrane-associated ring finger (C3HC4) 4 |
| BTRC | 1.21 | beta-transducin repeat containing, BU624603 |
| AQP5 | 1.21 | aquaporin 5 |
| PVALB | 1.21 | parvalbumin |
| MAMDC2 | 1.21 | MAM domain containing 2 |
| TGM1 | 1.21 | transglutaminase 1 |
| KCNB1 | 1.20 | potassium voltage-gated channel |
| COL25A1 | 1.20 | collagen, type XXV, alpha 1 |
| MRCL3 | 1.20 | myosin regulatory light chain 3 |
| KRTAP1-5 | 1.19 | keratin associated protein 1-5 |
| ROD1 | 1.19 | ROD1 regulator of differentiation 1 (S. pombe) |
| UAP1 | 1.19 | UDP-N-acteylglucosamine pyrophosphorylase 1 |
| PUSL1 | 1.19 | pseudouridylate synthase-like 1 |
| ACVRL1 | 1.18 | activin A receptor type II-like 1 |
| C21orf7 | 1.17 | chromosome 21 open reading frame 7 |
| GPR56 | 1.17 | G protein-coupled receptor 56 |
| GDF6PT | 1.17 | growth differentiation factor 6 |
| C9orf19 | 1.17 | chromosome 9 open reading frame 19 |
| KRTAP2-4 | 1.17 | keratin associated protein 2-4 |
| EIF5A2 | 1.16 | eukaryotic translation initiation factor 5A2 |
| CCND2 | 1.16 | cyclin D2 |
| CD164 | 1.16 | CD164 molecule, sialomucin |
| LIMS2 | 1.15 | LIM and senescent cell antigen-like domains 2 |
| C15orf39 | 1.15 | chromosome 15 open reading frame 39 |
| MEST | 1.14 | mesoderm specific transcript homolog (mouse) |
| PTPLB | 1.14 | protein tyrosine phosphatase-like |
| CEACAM19 | 1.14 | carcinoembryonic antigen-related |
| HSPA5 | 1.14 | heat shock 70kDa protein |
| NAP1L1 | 1.13 | nucleosome assembly protein 1-like 1 |
| PSG4 | 1.12 | pregnancy specific beta-1-glycoprotein 4 |
| MKNK2 | 1.12 | MAP kinase interacting serine/threonine kinase 2 |
| PNPLA3 | 1.12 | patatin-like phospholipase domain containing 3 |
| TTC9 | 1.12 | tetratricopeptide repeat domain 9 |
| EMD | 1.12 | emerin (Emery-Dreifuss muscular dystrophy) |
| AJAP1 | 1.11 | adherens junction associated protein 1, AK001007 |
| PDGFB | 1.11 | platelet-derived growth factor |
| CR603184 | 1.11 | complement component |
| PLAU | 1.11 | plasminogen activator, urokinase |
| TMEM16B | 1.11 | transmembrane protein 16B |
| LRRC17 | 1.10 | leucine rich repeat containing 17 |
| C8orf34 | 1.09 | chromosome 8 open reading frame 34 |
| HN1L | 1.08 | hematological and neurological expressed 1-like |
| MAP3K7 | 1.08 | protein kinase kinase kinase 7 |
| TMEM64 | 1.08 | transmembrane protein 64 |
| ANKS1A | 1.08 | ankyrin repeat sterile alpha motif 1A |
| ADRB2 | 1.08 | adrenergic, beta-2-, receptor, surface |
| SMURF2 | 1.07 | SMAD E3 ubiquitin protein ligase 2 |
| CHCHD6 | 1.06 | coiled-coil-helix-coiled-coil-helix domain |
| PQLC3 | 1.06 | PQ loop repeat containing 3 |
| SHC2 | 1.05 | SHC (Src homology 2 domain) |
| PCSK9 | 1.05 | proprotein convertase subtilisin |
| TFG | 1.05 | TRK-fused gene |
| POLR2K | 1.04 | polymerase (RNA) II polypeptide K, 7.0kDa |
| UBP1 | 1.04 | upstream binding protein 1 (LBP-1a) |
| TPM1 | 1.04 | tropomyosin 1 (alpha) |
| EPOR | 1.04 | erythropoietin receptor |
| SDF2L1 | 1.04 | stromal cell-derived factor 2-like 1 |
| PPP1R2 | 1.04 | protein phosphatase 1 |
| TNRC18 | 1.03 | trinucleotide repeat containing 18, KIAA1856 |
| CAMLG | 1.03 | calcium modulating ligand |
| ADAM19 | 1.03 | ADAM metallopeptidase domain 19 |
| PLEKHG2 | 1.03 | pleckstrin homology domain protein |
| MMP10 | 1.02 | matrix metallopeptidase 10 |
| C1orf174 | 1.02 | chromosome 1 open reading frame 174 |
| BFSP2 | 1.02 | beaded filament structural protein 2, phakinin, BG686022 |
| KRT19 | 1.02 | keratin 19 |
| TUFT1 | 1.02 | tuftelin 1 |
| MIER2 | 1.02 | mesoderm induction early response 1 |
| CBS | 1.01 | cystathionine-beta-synthase |
| CDK6 | 1.01 | cyclin-dependent kinase 6 |
| MEGF6 | 1.01 | multiple EGF-like-domains 6 |
| MARCH3 | 1.01 | membrane-associated ring finger (C3HC4) 3 |
| CD3EAP | 1.01 | CD3e molecule, epsilon associated protein |
| PAQR5 | 1.01 | progestin and adipoQ receptor member V |
| TUBB3 | 1.01 | tubulin, beta 3 |
| NDST1 | 1.01 | N-deacetylase/N-sulfotransferase |
| MUC1 | 1.01 | mucin 1, cell surface associated |
| BASP1 | 1.00 | (brain) membrane attached signal protein 1 |
| ISOC2 | 1.00 | isochorismatase domain containing 2 |
| FGF2 | 1.00 | fibroblast growth factor 2 (basic) |
| RGS9 | 1.00 | regulator of G-protein signalling 9 |
| MCF2L | 0.99 | MCF.2 cell line derived transforming sequence-like |
| GAS2L1 | 0.99 | growth arrest-specific 2 like 1 |
| GNPNAT1 | 0.99 | glucosamine-phosphate N-acetyltransferase 1 |
| RIMBP3 | 0.99 | RIMS binding protein 3, KIAA1666 |
| FAM90A10 | 0.99 | family with sequence similarity 90, member A10 |
| FABP3 | 0.99 | fatty acid binding protein 3, muscle and heart |
| ACTB | 0.99 | actin, beta |
| COL8A2 | 0.99 | collagen, type VIII, alpha 2 |
| RIMS3 | 0.99 | regulating synaptic membrane exocytosis 3 |
| PCK2 | 0.99 | phosphoenolpyruvate carboxykinase 2 (mitochondrial) |
| ARPC1B | 0.99 | actin related protein 2/3 complex, subunit 1B, 41kDa |
| JUP | 0.98 | junction plakoglobin |
| RGC32 | 0.98 | chr13 orf 15, thought to regulate cell cycle progression |
| KRTAP1-1 | 0.98 | keratin associated protein 1-1 |
| HES2 | 0.98 | hairy and enhancer of split 2 (Drosophila) |
| PPME1 | 0.97 | protein phosphatase methylesterase 1 |
| MAP6 | 0.97 | microtubule-associated protein 6 |
| IGFBP3 | 0.97 | insulin-like growth factor binding protein 3 |
| FLNB | 0.97 | Filamin B |
| TNFRSF12A | 0.97 | tumor necrosis factor receptor superfamily, member 12A |
| C14orf80 | 0.97 | chromosome 14 open reading frame 80 |
| LHFPL2 | 0.96 | lipoma HMGIC fusion partner-like 2 |
| ZDHHC20 | 0.96 | zinc finger, DHHC-type containing 20 |
| DERL3 | 0.96 | Der1-like domain family, member 3 |
| OSBPL6 | 0.96 | oxysterol binding protein-like 6 |
| CHST2 | 0.96 | carbohydrate (N-acetylglucosamine-6-O) sulfotransferase 2 |
| TMEM51 | 0.95 | transmembrane protein 51 |
| NTF3 | 0.95 | neurotrophin 3 |
| ESYT2 | 0.95 | extended synaptotagmin-like protein 2, FAM62B |
| NLRP1 | 0.95 | NLR family, pyrin domain containing 1 |
| CLDND1 | 0.95 | claudin domain containing 1 |
| YRDC | 0.95 | yrdC domain containing (E. coli) |
| LONRF1 | 0.94 | LON peptidase N-terminal domain and ring finger 1 |
| BCR | 0.94 | breakpoint cluster region |
| SPCS3 | 0.94 | signal peptidase complex subunit 3 homolog |
| RUNX1 | 0.94 | runt-related transcription factor 1 |
| NFAT5 | 0.94 | nuclear factor of activated T-cells 5, tonicity-responsive |
| RAI14 | 0.93 | retinoic acid induced 14 |
| SNN | 0.93 | stannin |
| TUBA3D | 0.93 | tubulin, alpha 3d;H2-alpha |
| ZNF148 | 0.93 | zinc finger protein 148 |
| BAIAP2 | 0.93 | BAI1-associated protein 2 |
| SH2D5 | 0.93 | SH2 domain containing 5 |
| DAB2 | 0.93 | disabled homolog 2, mitogen-responsive phosphoprotein |
| ODF2 | 0.92 | outer dense fiber of sperm tails 2 |
| DENND5A | 0.92 | DENN/MADD domain containing 5A, RAB6IP1 |
| TBC1D10A | 0.92 | TBC1 domain family, member 10A |
| KRT33A | 0.92 | keratin 33A |
| BCAS4 | 0.92 | breast carcinoma amplified sequence 4 |
| AGPAT7 | 0.92 | lysophosphatidylcholine acyltransferase 4 |
| NAV2 | 0.92 | neuron navigator 2 |
| GRAMD3 | 0.91 | GRAM domain containing 3 |
| TUBB8 | 0.91 | tubulin, beta 8 |
| LOXL2 | 0.91 | lysyl oxidase-like 2 |
| ZC3HAV1L | 0.91 | zinc finger CCCH-type, antiviral 1-like |
| MALL | 0.91 | mal, T-cell differentiation protein-like |
| PEA15 | 0.91 | phosphoprotein enriched in astrocytes 15 |
| CSRP1 | 0.91 | cysteine and glycine-rich protein 1 |
| CDK2AP2 | 0.91 | cyclin-dependent kinase 2 associated protein 2 |
| STYK1 | 0.90 | serine/threonine/tyrosine kinase 1 |
| DPYSL5 | 0.90 | dihydropyrimidinase-like 5 |
| HOMER2 | 0.90 | homer homolog 2 (Drosophila) |
| GCS1 | 0.90 | mannosyl-oligosaccharide glucosidase |
| PPP1R1A | 0.90 | protein phosphatase 1, regulatory (inhibitor) subunit 1A |
| SLC7A7 | 0.90 | solute carrier family 7 |
| PYGB | 0.90 | phosphorylase, glycogen; brain |
| CLIC4 | 0.90 | chloride intracellular channel 4 |
| GBE1 | 0.90 | glucan (1,4-alpha-), branching enzyme 1 |
| EXPH5 | 0.89 | exophilin 5 |
| BDNF | 0.89 | brain-derived neurotrophic factor |
| ACTN4 | 0.89 | actinin, alpha 4 |
| SQLE | 0.88 | squalene epoxidase |
| CCL2 | 0.88 | chemokine (C-C motif) ligand 2 |
| CCND1 | 0.88 | cyclin D1 |
| SETD7 | 0.88 | SET domain containing (lysine methyltransferase) 7 |
| FLJ38359 | 0.88 | hypothetical LOC151009 |
| TRIOBP | 0.88 | TRIO and F-actin binding protein |
| ADAMTS14 | 0.88 | ADAM metallopeptidase with thrombospondin type 1 motif, 14 |
| TBCD | 0.88 | tubulin folding cofactor D |
| PRKAA2 | 0.88 | protein kinase, AMP-activated, alpha 2 catalytic subunit |
| NEK7 | 0.88 | NIMA (never in mitosis gene a)-related kinase 7 |
| NDUFC2 | 0.88 | NADH dehydrogenase (ubiquinone) |
| SLC3A2 | 0.88 | solute carrier family 3 |
| BBS9 | 0.88 | Bardet-Biedl syndrome 9, PTHB1 |
| SLC20A2 | 0.88 | solute carrier family 20 (phosphate transporter), member 2 |
| LDLR | 0.87 | low density lipoprotein receptor |
| SELIL3 | 0.87 | sel-1 suppressor of lin-12-like 3 (C. elegans), KIAA0746 |
| B3GALT4 | 0.87 | UDP-Gal:betaGlcNAc beta 1,3-galactosyltransferase, poly 4 |
| ARID5A | 0.87 | AT rich interactive domain 5A (MRF1-like) |
| UNC119 | 0.87 | unc119 (C.elegans) homolog |
| ADAM12 | 0.87 | alpha disintegrin and metalloproteinase domain 12 (meltrin alpha) |
| SLC7A5 | 0.87 | large neutral amino acids transporter small subunit 1 |
| LOC338620 | 0.87 | hypothetical protein LOC338620 |
| MFAP5 | 0.87 | microfibrillar associated protein 5 |
| PRR13 | 0.86 | proline-rich protein 13 |
| TUBA3 | 0.86 | tubulin, alpha 3 |
| KCNJ15 | 0.86 | Inward rectifier K(+) channel Kir1.3 |
| SUDS3 | 0.86 | sin3A-associated protein, 45kDa |
| DENND2A | 0.86 | DENN/MADD domain containing 2A |
| TIMP3 | 0.86 | metalloproteinase inhibitor 3 |
| PJCG6 | 0.86 | olfactory receptor, family 7, subfamily E |
| ANXA2 | 0.86 | annexin-2 |
| RAB3B | 0.86 | ras-related protein Rab-3B |
| PPP1R13L | 0.86 | inhibitor of apoptosis stimulating protein of p53 |
| IBRDC2 | 0.86 | IBR domain-containing protein 2, ring finger protein 144B |
| DHRS3 | 0.86 | short-chain dehydrogenase/reductase 1 |
| ACBD5 | 0.86 | acyl-Coenzyme A binding domain containing 5 |
| MFN2 | 0.85 | transmembrane GTPase MFN2 |
| DNAJB6 | 0.85 | DnaJ (Hsp40) homolog, subfamily B, member 6 |
| MYADM | 0.85 | myeloid-associated differentiation marker |
| KRT15 | 0.85 | keratin, type I cytoskeletal 15 |
| NPNT | 0.85 | nephronectin |
| ADAMTSL5 | 0.85 | thrombospondin type-1 domain-containing protein 6 |
| STAU2 | 0.85 | staufen, RNA binding protein, homolog 2 |
| FAM49A | 0.85 | family with sequence similarity 49, member A |
| KCNK6 | 0.85 | potassium channel subfamily K member 6 |
| CHST3 | 0.84 | carbohydrate sulfotransferase 3 |
| CDR2L | 0.84 | paraneoplastic antigen |
| AMMECR1 | 0.84 | AMME syndrome candidate gene 1 protein |
| GPSM1 | 0.84 | activator of G-protein signaling 3 |
| COTL1 | 0.84 | coactosin-like protein |
| HMGA2 | 0.84 | high mobility group protein HMGI-C |
| TUBB | 0.83 | beta 5-tubulin |
| CRMP1 | 0.83 | collapsin response mediator protein 1, dihydropyrimidinase-like 1 |
| CXorf39 | 0.83 | chromosome X open reading frame 39 |
| F13A1 | 0.83 | transglutaminase A chain |
| HNRPAB | 0.82 | hnRNP type A/B protein |
| OXSR1 | 0.82 | oxidative stress-responsive 1 protein |
| GADD45B | 0.82 | growth arrest and DNA-damage-inducible, beta |
| IPO5 | 0.82 | Importin subunit beta-3 , RANBP5 |
| SPINT2 | 0.82 | hepatocyte growth factor activator inhibitor type 2 |
| BTBD7 | 0.82 | |BTB/POZ domain-containing protein 7 |
| ARHGAP23 | 0.82 | rho-type GTPase-activating protein 23 |
| ACPL2 | 0.81 | acid phosphatase-like protein 2 |
| LOC442421 | 0.81 | hypothetical LOC442421 |
| COPB1 | 0.81 | coatomer protein complex, subunit beta 1 |
| CYYR1 | 0.81 | cysteine/tyrosine-rich 1 |
| DIXDC1 | 0.81 | Coiled-coil-DIX1 |
| TMAP1 | 0.81 | Transmembrane anchor protein 1, MXRA7 |
| SIRPA | 0.81 | MYD-1 , signal-regulatory protein alpha-2 |
| METRNL | 0.81 | meteorin-like protein |
| TRIML2 | 0.81 | SPRY domain-containing protein 6, FLJ25801 |
| CHST7 | 0.80 | Chondroitin 6-sulfotransferase 2 |
| PID1 | 0.80 | phosphotyrosine interaction domain containing 1, FLJ20701 |
| ANKRD54 | 0.80 | ankyrin repeat domain-containing protein 54 |
| CNN2 | 0.80 | Calponin H2, smooth muscle |
| AADACL1 | 0.80 | Arylacetamide deacetylase-like 1 |
| ARHGEF4 | 0.80 | Rho guanine nucleotide exchange factor (GEF) 4 |
| CHRD | 0.80 | chordin |
