# Supplementary material for: Multi-Tasking Role of the Mechanosensing Protein Ankrd2 in the Signaling Network of Striated Muscle
Source: PLoS One. 2011 Oct 10;6(10):e25519. doi: 10.1371/journal.pone.0025519 (PMC3189947; doi:10.1371/journal.pone.0025519)
Supplement: Table S3 — Differentially expressed genes in infected (non silenced) compared to uninfected CHQ5B cells. (DOC) [file pone.0025519.s005.doc]

**Table S3. Differentially expressed genes in infected (non silenced) compared to uninfected CHQ5B cells*.**

| Gene symbol | Log2 ratio | Gene symbol | Log2 ratio | Gene symbol | Log2 ratio |
| --- | --- | --- | --- | --- | --- |
| BIRC5 | -3.98 | ANLN | -1.73 | DTL | -1.42 |
| KIAA0101 | -3.38 | KCNE1L | -1.71 | GPRC5C | -1.41 |
| UBE2C | -3.36 | C15orf42 | -1.71 | PLAT | -1.41 |
| NCAPG | -3.33 | TROAP | -1.70 | A23P21882 | -1.39 |
| PBK | -3.29 | SCT | -1.69 | BC007606 | -1.39 |
| KNTC2 | -3.13 | RP11217H1.1 | -1.68 | LOXL2 | -1.39 |
| FAM64A | -3.020 | KIF15 | -1.66 | EXO1 | -1.39 |
| CDC45L | -2.96 | CTSH | -1.66 | TMEM51 | -1.39 |
| CDCA8 | -2.68 | NCAPH | -1.64 | AQP5 | -1.38 |
| CENPF | -2.61 | ADAM12 | -1.63 | ADAMTS4 | -1.38 |
| NUF2 | -2.60 | LOC387763 | -1.63 | WNT10A | -1.38 |
| CDT1 | -2.55 | RAD54L | -1.63 | STMN2 | -1.36 |
| CDC2 | -2.55 | HMMR | -1.62 | TSPYL4 | -1.36 |
| DEFB103A | -2.42 | C9orf58 | -1.61 | NUSAP1 | -1.36 |
| F13A1 | -2.39 | SHCBP1 | -1.61 | FANCA | -1.36 |
| MAL | -2.38 | CDCA7 | -1.61 | ATP8B2 | -1.36 |
| CCNB2 | -2.37 | RAB15 | -1.60 | KIF20A | -1.35 |
| CEP55 | -2.29 | PSG1 | -1.59 | MAD2L1 | -1.35 |
| GPR56 | -2.27 | CNN1 | -1.59 | MDH1 | -1.34 |
| TOP2A | -2.24 | RASSF2 | -1.58 | RASSF7 | -1.34 |
| KISS1 | -2.17 | HSPA5 | -1.58 | FANCD2 | -1.33 |
| PKMYT1 | -2.16 | SPRR2G | -1.57 | ATP10A | -1.33 |
| SPBC25 | -2.15 | CCL2 | -1.57 | CARD10 | -1.32 |
| MYOZ2 | -2.14 | PVALB | -1.56 | RECQL4 | -1.32 |
| ASPM | -2.14 | CCNB1 | -1.55 | CDK6 | -1.30 |
| CDCA5 | -2.12 | RAD51AP1 | -1.55 | PSMC3IP | -1.30 |
| E2F2 | -2.11 | TNFRSF25 | -1.55 | PPP1R2 | -1.30 |
| GNAZ | -2.10 | CIT | -1.53 | SPAG5 | -1.29 |
| A24P110601 | -2.05 | ISOC1 | -1.53 | UHMK1 | -1.29 |
| PLAU | -2.04 | PACSIN1 | -1.52 | EML1 | -1.29 |
| DLG7 | -2.00 | BUB1 | -1.51 | GALNTL1 | -1.29 |
| OIP5 | -1.98 | DERL3 | -1.50 | CAV1 | -1.29 |
| C13orf3 | -1.96 | GRASP | -1.50 | FOXM1 | -1.29 |
| TK1 | -1.95 | HN1L | -1.50 | CLIC4 | -1.29 |
| PRC1 | -1.95 | MPP4 | -1.50 | C1orf38 | -1.28 |
| CRIP1 | -1.93 | MELK | -1.49 | A32P171043 | -1.28 |
| KIF2C | -1.91 | KIF23 | -1.49 | E2F1 | -1.28 |
| RRM2 | -1.90 | CDC6 | -1.48 | GPC6 | -1.28 |
| TMSL8 | -1.90 | MLF1IP | -1.48 | ZWINT | -1.28 |
| RAD51 | -1.86 | SQLE | -1.46 | AF086187 | -1.28 |
| NANOS1 | -1.82 | TMEM64 | -1.46 | HTR3E | -1.28 |
| CDCA2 | -1.82 | PLEKHG5 | -1.46 | EFNA1 | -1.28 |
| GINS2 | -1.80 | UHRF1 | -1.44 | RACGAP1 | -1.27 |
| KRT80 | -1.79 | PDGFRB | -1.44 | PLK1 | -1.26 |
| DMBT1 | -1.77 | PUSL1 | -1.43 | DKFZp762E1312 | -1.25 |
| SFRP4 | -1.75 | CD164 | -1.42 |  |  |

*All 137 genes selected as significant with 5% FDR by the SAM software are reported. In bold italics are the 13 genes identified by the same software as significantly regulated with 0% FDR.
