# Supplementary material for: Multi-Tasking Role of the Mechanosensing Protein Ankrd2 in the Signaling Network of Striated Muscle
Source: PLoS One. 2011 Oct 10;6(10):e25519. doi: 10.1371/journal.pone.0025519 (PMC3189947; doi:10.1371/journal.pone.0025519)
Supplement: Table S4 — KEGG pathways with 7 or more genes differentially expressed in Ankrd2 silenced myotubes. (DOC) [file pone.0025519.s006.doc]

| **Table S4. KEGG pathways with 7 or more genes differentially expressed  in Ankrd2 silenced myotubes** | | | |
| --- | --- | --- | --- |
| **Gene Symbol** | **Gene Description** | | **Log2 Ratio** |
| **hsa01100 Metabolic pathways - Homo sapiens (34)** | | | |
| ADSSL1 | adenylosuccinate synthase like 1, FLJ38602 | | - 0.98 |
| AKR1B10 | aldo-keto reductase family 1 | | - 0.99 |
| AMPD1 | adenosine monophosphate deaminase 1 | | - 1.95 |
| AMY1C | amylase, alpha 1C | | - 0.86 |
| ATP6V1E2 | ATPase, H+ transporting, V1 subunit E2 | | - 0.81 |
| BCAT1 | branched chain amino-acid transaminase 1, cytosolic | | - 0.99 |
| B3GALT4 | UDP-Gal:betaGlcNAc beta 1,3-galactosyltransferase, polypeptide 4 | | + 0.87 |
| CBS | cystathionine-beta-synthase | | + 1.01 |
| CKM | creatine kinase, muscle | | - 0.82 |
| CYP27A1 | cytochrome P450, family 27, subfamily A, polypeptide 1 | | - 0.82 |
| DHRS3 | dehydrogenase/reductase (SDR family) member 3 | | + 0.86 |
| GBE1 | glucan (1,4-alpha-), branching enzyme 1 | | + 0.90 |
| GCNT3 | N-acetylglucosaminyltransferase 3, mucin type | | - 1.23 |
| GCS1 | mannosyl-oligosaccharide glucosidase ;MOGS | | + 0.90 |
| GLUL | glutamate-ammonia ligase | | - 0.82 |
| HADH | hydroxyacyl-CoA dehydrogenase | | - 1.14 |
| KHK | ketohexokinase (fructokinase) | | + 1.38 |
| MLYCD | malonyl-CoA decarboxylase | | - 1.01 |
| NDST1 | N-deacetylase/N-sulfotransferase (heparan glucosaminyl) 1 | | + 1.01 |
| NDUFC2 | NADH dehydrogenase (ubiquinone) 1 subcomplex unknown 2 | | + 0.88 |
| PCK2 | phosphoenolpyruvate carboxykinase 2 (mitochondrial) | | + 0.99 |
| PFKM | phosphofructokinase, muscle | | - 0.92 |
| PIK3C2B | phosphoinositide-3-kinase, class 2, beta polypeptide | | - 1.18 |
| PLCB4 | phospholipase C, beta 4 | | + 1.26 |
| PLCD4 | phospholipase C, delta 4 | | - 0.84 |
| PNPLA3 | patatin-like phospholipase domain-containing protein 3 | | + 1.12 |
| POLR2K | polymerase (RNA) II (DNA directed) polypeptide K | | + 1.04 |
| PPT1 | palmitoyl-protein thioesterase 1 | | - 1.17 |
| SC4MOL | sterol-C4-methyl oxidase-like | | + 1.23 |
| SPTLC3 | serine palmitoyltransferase, long chain base subunit 3 | | - 0.86 |
| SQLE | squalene epoxidase | | + 0.88 |
| ST8SIA5 | ST8 alpha-N-acetyl-neuraminide alpha-2,8-sialyltransferase 5 | | - 0.97 |
| TRIT1 | tRNA isopentenyltransferase 1 | | - 0.80 |
| UAP1 | UDP-N-acetylglucosamine pyrophosphorylase | | + 1.19 |
|  | | | |
| **hsa05200 pathways in cancer - Homo sapiens (17)** | | | |
| ARNT | aryl hydrocarbon receptor (HIF1B) | | - 0.90 |
| BCR | breakpoint cluster region (ABL) | | + 0.94 |
| CCND1 | cyclin D1 | | + 0.88 |
| CDK6 | cyclin-dependent kinase 6 | | + 1.01 |
| CYCS | cytochrome c, somatic | | - 1.04 |
| EGLN3 | egl nine homolog 3 (HPH, PHD3) | | - 1.41 |
| FGF2 | fibroblast growth factor 2 (basic) | | + 1.10 |
| FZD4 | frizzled homolog 4 | | - 1.14 |
| ITGA6 | integrin, alpha 6 | | + 1.65 |
| JUP | junction plakoglobin | | + 0.98 |
| LAMA4 | laminin, alpha 4 | | - 0.81 |
| MAP2K1 | mitogen-activated protein kinase kinase 1 | | + 1.38 |
| PDGFB | platelet-derived growth factor beta polypeptide | | + 1.11 |
| PDGFRB | platelet-derived growth factor receptor, beta polypeptide | | + 1.77 |
| RUNX1 | runt-related transcription factor 1 | | + 0.94 |
| TFG | TRK-fused gene; protein TFG | | + 1.05 |
| TGFB2 | transforming growth factor, beta 2 | | + 1.41 |
|  | | | |
| **hsa04510 Focal adhesion - Homo sapiens (14)** | | | |
| ACTB | actin beta/gamma 1 | | + 0.99 |
| ACTN4 | actinin, alpha 4 | | + 0.89 |
| CAV1 | caveolin 1, caveolae protein, 22kDa | | + 1.41 |
| CCND1 | cyclin D1 | | + 0.88 |
| CCND2 | cyclin D2 | | + 1.16 |
| FLNB | filamin B, beta (actin binding protein 278) | | + 0.97 |
| ITGA6 | Integrin , alpha 6 | | + 1.65 |
| ITGB8 | integrin, beta 8 | | - 1.26 |
| LAMA4 | laminin, alpha 4 | | - 0.81 |
| MAP2K1 | mitogen-activated protein kinase kinase 1 (MEK1) | | + 1.38 |
| MYL5 | myosin, light chain 5, regulatory | | - 0.89 |
| PDGFB | platelet-derived growth factor beta polypeptide | | + 1.11 |
| PDGFRB | platelet-derived growth factor receptor, beta polypeptide | | + 1.77 |
| SHC2 | SHC (Src homology 2 domain containing) transforming protein 2 | | + 1.05 |
|  | | | |
| **hsa04010 MAPK signaling pathway - Homo sapiens (13)** | | | |
| BDNF | brain-derived neurotrophic factor | | + 0.89 |
| FGF2 | fibroblast growth factor 2 (basic) | | + 1.00 |
| FLNB | filamin B | | + 0.97 |
| GADD45B | growth arrest and DNA-damage-inducible, beta | | + 0.82 |
| MAP2K1 | mitogen-activated protein kinase kinase 1 (MEK1) | | + 1.38 |
| MAP3K7 | mitogen-activated protein kinase kinase kinase 7 (TAK1) | | + 1.08 |
| MEF2C | myocyte enhancer factor 2C | | - 1.08 |
| MKNK2 | MAP kinase interacting serine/threonine kinase 2 | | + 1.12 |
| NTF3 | neurotrophin 3 | | + 0.95 |
| PDGFB | platelet-derived growth factor beta polypeptide | | + 1.11 |
| PDGFRB | platelet-derived growth factor receptor, beta polypeptide | | + 1.77 |
| RPS6KA5 | ribosomal protein S6 kinase, 90kDa, polypeptide 5 | | - 0.91 |
| TGFB2 | transforming factor , beta 2 | | + 1.41 |
|  | | | |
| **hsa04060 Cytokine-cytokine receptor interaction - Homo sapiens (12)** | | | |
| ACVR1 | activin A receptor, type I | | - 0.85 |
| CCL2 | chemokine (C-C motif) ligand 2 | | + 0.88 |
| EPOR | erythropoietin receptor | | + 1.04 |
| IL17B | interleukin 17B | | - 0,99 |
| IL6R | interleukin 6 receptor | | - 0.81 |
| NGFR | nerve growth factor receptor | | + 1.88 |
| PDGFB | platelet-derived growth factor beta polypeptide | | + 1.11 |
| PDGFRB | platelet-derived growth factor receptor, beta polypeptide | | + 0.97 |
| TGFB2 | transforming growth factor, beta 2 | | + 1.41 |
| TNFRSF11B | Tumor necrosis factor receptor superfamily, member 11b | | + 0.89 |
| TNFRSF12A | tumor necrosis factor receptor superfamily, member 12A | | + 0.97 |
| TNFRSF25 | tumor necrosis factor receptor superfamily, member 25 | | + 1.69 |
|  | | | |
| **hsa04810 Regulation of actin cytoskeleton- Homo sapiens (12)** | | | |
| ACTB | Actin, beta | | + 0.99 |
| ACTN4 | actinin, alpha 4 | | + 0.89 |
| ARHGEF4 | Rho guanine nucleotide exchange factor (GEF) 4 | | + 0.80 |
| ARPC1B | actin related protein 2/3 complex, subunit 1B, 41kDa | | + 0.99 |
| BAIAP2 | BAI1-associated protein 2 | | + 0.93 |
| FGF2 | fibroblast growth factor 2 (basic) | | + 1.00 |
| ITGA6 | integrin, alpha 6 | | +1.64 |
| ITGB8 | integrin, beta 8 | | - 1 .26 |
| MAP2K1 | mitogen-activated protein kinase kinase 1 (MEK1) | | + 1.38 |
| MYL5 | myosin, light chain 5, regulatory | | - 0.89 |
| PDGFB | platelet-derived growth factor beta polypeptide | | + 1.11 |
| PDGFRB | platelet-derived growth factor receptor, beta polypeptide | | + 1.77 |
|  | | | |
| **hsa04910 Insulin signaling pathway- Homo sapiens (12)** | | | |
| MAP2K1 | mitogen-activated protein kinase kinase 1 | | + 1.38 |
| MKNK2 | MAP kinase interacting serine/threonine kinase 2 | | + 1.12 |
| PCK2 | phosphoenolpyruvate carboxykinase 2 (mitochondrial) | | + 0.99 |
| PHKG1 | phosphorylase kinase, gamma 1 (muscle) | | - 1.74 |
| PPARGC1A | peroxisome proliferator-activated receptor gamma | | - 1.61 |
| PRKAA2 | protein kinase, AMP-activated, alpha 2 catalytic subunit | | + 0.88 |
| PRKAG2 | protein kinase, AMP-activated, gamma 2 non-catalytic subunit | | + 1.26 |
| PTPRF | protein tyrosine phosphatase, receptor type, F | | + 1.22 |
| PYGB | phosphorylase, glycogen; brain; starch phosphorylase | | + 0.90 |
| PYGM | phosphorylase, glycogen, muscle | | - 1.15 |
| RPS6KB1 | ribosomal protein S6 kinase, 70kDa | | - 0.82 |
| SHC2 | SHC (Src homology 2 domain containing) transforming protein 2 | | + 1.05 |
|  | | | |
| **hsa04310 Wnt signaling pathway- Homo sapiens (10)** | | | |
| CCND1 | cyclin D1 | | + 0.88 |
| CCND2 | cyclin D2 | | + 1.16 |
| DAAM1 | dishevelled associated activator of morphogenesis 1 | | - 0.89 |
| FRAT2 | frequently rearranged in advanced T-cell lymphomas 2 | | - 1.15 |
| FZD4 | frizzled homolog 4 (Drosophila) | | - 1.14 |
| MAP3K7 | mitogen-activated protein kinase kinase kinase 7 | | + 1.08 |
| MMP7 | matrix metallopeptidase 7 (matrilysin, uterine) | | - 1.01 |
| NFAT5 | nuclear factor of activated T-cells 5, calcineurin-dependent | | + 0.94 |
| PLCB4 | phospholipase C, beta 4 | | + 1.26 |
| SFRP4 | secreted frizzled-related protein 4 | | + 2.24 |
|  | | | |
| **hsa04020 Calcium signaling pathway - Homo sapiens (9)** | | | |
| ADCY3 | adenylate cyclase 3 | | - 0.90 |
| ADRA1B | adrenergic, alpha-1B-, receptor | | + 1.25 |
| ADRB2 | adrenergic, beta-2-, receptor, surface | | + 1.08 |
| ATP2A1 | ATPase, Ca++ transporting, cardiac muscle, fast twitch 1 | | - 1.51 |
| PDGFRB | platelet-derived growth factor receptor, beta polypeptide | | + 1.77 |
| PHKG1 | phosphorylase kinase, gamma 1 (muscle) | | - 1.74 |
| PLCB4 | phospholipase C, beta 4 | | + 1.26 |
| PLCD4 | phospholipase C, delta 4 | | - 0.84 |
| TNNC2 | troponin C type 2 (fast); troponin C, skeletal muscle | | - 1.11 |
|  | | | |
| **hsa04540 Gap junction- Homo sapiens (9)** | | | |
| ADCY3 | adenylate cyclase 3 | | - 0.90 |
| MAP2K1 | mitogen-activated protein kinase kinase 1 | | + 1.38 |
| PDGFB | platelet-derived growth factor beta polypeptide | | + 1.11 |
| PDGFRB | platelet-derived growth factor receptor, beta polypeptide | | + 1.77 |
| PLCB4 | phospholipase C, beta 4 | | + 1.26 |
| TUBA8 | tubulin, alpha 8 | | - 1.00 |
| TUBB | tubulin, beta | | + 0.83 |
| TUBB8 | tubulin, beta 8 | | + 0.91 |
| TUBB3 | tubulin, beta 3 | | + 1.01 |
|  | | | |
| **hsa05410 Hypertrophic cardiomyopathy (HCM) - Homo sapiens (8)** | | | |
| ACTB | Actin, beta | | + 0.99 |
| EMD | emerin | | +1.12 |
| ITGA6 | integrin, alpha 6 | | +1.64 |
| PRKAA2 | protein kinase, AMP-activated, alpha 2 catalytic subunit | | + 0.88 |
| PRKAG2 | protein kinase, AMP-activated, gamma 2 non-catalytic subunit | | + 1.26 |
| TGFB2 | transforming factor , beta 2 | | + 1.41 |
| TPM1 | tropomyosin 1 (alpha) | | + 1.04 |
| TTN | titin | | - 1.03 |
|  | | | |
| **hsa05414 Dilated cardiomyopathy (DCM) - Homo sapiens (human) (7)** | | | |
| ACTB | Actin, beta | | + 0.99 |
| ADCY3 | adenylate cyclase 3 | | - 0.90 |
| EMD | emerin | | +1.12 |
| ITGA6 | integrin, alpha 6 | | +1.64 |
| TGFB2 | transforming factor , beta 2 | | + 1.41 |
| TPM1 | tropomyosin 1 (alpha) | | + 1.04 |
| TTN | titin | | - 1.03 |
|  | | | |
| **hsa05220 Chronic myeloid leukemia - Homo sapiens (7)** | | | |
| BCR | breakpoint cluster region (ABL) | | + 0.94 |
| CCND1 | cyclin D1 | | + 0.88 |
| CDK6 | cyclin-dependent kinase 6 | | + 1.01 |
| MAP2K1 | mitogen-activated protein kinase kinase 1 | | + 1.38 |
| RUNX1 | runt-related transcription factor 1 | | + 0.94 |
| SHC2 | Src homology 2 domain containing) transforming protein 2 | | + 0.93 |
| TGFB2 | transforming growth factor, beta 2 | | + 1.41 |
|  | | | |
| **hsa04144 Endocytosis - Homo sapiens (7)** | | | |
| ADRB2 | adrenergic, beta-2-, receptor, surface | | + 1.08 |
| CAV1 | caveolin 1, caveolae protein, 22kDa | | + 1.41 |
| DAB2 | disabled homolog 2, mitogen-responsive phosphoprotein | | + 0.93 |
| FOLR1 | folate receptor 1 (adult) | | + 1.39 |
| LDLR | low density lipoprotein receptor | | + 0.87 |
| SMURF2 | SMAD specific E3 ubiquitin protein ligase 2 | | + 1.07 |
| TGFB2 | transforming growth factor, beta 2 | | + 1.41 |
|  | | | |
| **hsa05016 Huntington's disease - Homo sapiens (human)(7)** | | | |
| BDNF | brain-derived neurotrophic factor; K04355 | | + 0.89 |
| CYCS | cytochrome c, somatic; K08738 cytochrome c | | - 1.04 |
| Dynein | dynein | | - 0.81 |
| NDUFC2 | NADH dehydrogenase (ubiquinone) | | + 0.88 |
| PLCB4 | phospholipase C, beta 4 | | + 1.26 |
| POLR2K | DNA-directed RNA Polymerase II subunit K | | + 1.04 |
| PPARGC1A | peroxisome proliferative activated receptor, gamma, coact. 1, alpha | | - 1.61 |
|  | | | |
| **hsa04115 p53 signaling pathway- Homo sapiens (7)** | | | |
| CCND1 | cyclin D1 | + 0.88 | |
| CCND2 | cyclin D2 | + 1.16 | |
| CDK6 | cyclin-dependent kinase 6 | + 1.01 | |
| CYCS | cytochrome c, somatic | - 1.04 | |
| GADD45B | growth arrest and DNA-damage-inducible, beta | + 0.82 | |
| IGFBP3 | serpin peptidase inhibitor | + 0.97 | |
| SERPINE1 | plasminogen activator inhibitor-1 | + 1.26 | |
|  | | | |
| **hsa04350 TGF-beta signaling pathway- Homo sapiens (7)** | | | |
| ACVR1 | activin A receptor, type I | | - 0.85 |
| CHRD | chordin | | + 0.80 |
| GDF6 | growth differentiation factor 6 | | + 1.17 |
| PITX2 | paired-like homeodomain transcription factor 2 | | - 1.07 |
| RPS6KB1 | ribosomal protein S6 kinase, 70kDa, polypeptide 1 | | - 0.82 |
| SMURF2 | SMAD specific E3 ubiquitin protein ligase 2 | | + 1.07 |
| TGFB2 | transforming growth factor, beta 2 | | + 1.41 |
|  | | | |
| **hsa04530 Tight junction - Homo sapiens (7)** | | | |
| ACTB | Actin, beta | | + 0.99 |
| ACTN4 | actinin, alpha 4 | | + 0.89 |
| MYH8 | myosin, heavy chain 8, skeletal muscle, perinatal | | - 0.83 |
| MYL5 | myosin, light chain 5, regulatory; | | - 0.89 |
| RAB3B | RAB3B, member RAS oncogene family; | | + 0.86 |
| TJP2 | tight junction protein 2 (zona occludens 2) | | - 1.16 |
| YES1 | v-yes-1 Yamaguchi sarcoma viral oncogene homolog 1 | | + 1.39 |
